# Evidence-based clinical practice guidelines for irritable bowel syndrome 2020

**DOI:** 10.1007/s00535-020-01746-z

**Published:** 2021-02-04

**Authors:** Shin Fukudo, Toshikatsu Okumura, Masahiko Inamori, Yusuke Okuyama, Motoyori Kanazawa, Takeshi Kamiya, Ken Sato, Akiko Shiotani, Yuji Naito, Yoshiko Fujikawa, Ryota Hokari, Tastuhiro Masaoka, Kazuma Fujimoto, Hiroshi Kaneko, Akira Torii, Kei Matsueda, Hiroto Miwa, Nobuyuki Enomoto, Tooru Shimosegawa, Kazuhiko Koike

**Affiliations:** 1 Guidelines Committee for Creating and Evaluating the “Evidence-Based Clinical Practice Guidelines for Irritable Bowel Syndrome”, The Japanese Society of Gastroenterology, 6F Shimbashi i-MARK Building, 2-6-2 Shimbashi, Minato-ku, Tokyo 105-0004 Japan; 2 Department of Behavioral Medicine Tohoku University Graduate School of Medicine, 2-1 Seiryo-machi, Aoba-ku, Sendai, 980-8575 Japan

**Keywords:** Antibiotics, Antidepressant, Brain-gut interactions, Complications, Diagnosis, Epidemiology, Functional bowel disorder (FBD), Functional gastrointestinal disorders (FGIDs), 5-HT3 antagonists, 5-HT4 agonists, Irritable bowel syndrome (IBS), Infection, Inflammation, Intestinal secretagogues (gut epithelial modifier), Microbiota, Mucosal permeability, Pathophysiology, Probiotics, Prognosis, Psychosocial stress, Psychotherapy, Rome IV criteria, Treatment

## Abstract

Managing irritable bowel syndrome (IBS) has attracted international attention because single-agent therapy rarely relieves bothersome symptoms for all patients. The Japanese Society of Gastroenterology (JSGE) published the first edition of evidence-based clinical practice guidelines for IBS in 2015. Much more evidence has accumulated since then, and new pharmacological agents and non-pharmacological methods have been developed. Here, we report the second edition of the JSGE-IBS guidelines comprising 41 questions including 12 background questions on epidemiology, pathophysiology, and diagnostic criteria, 26 clinical questions on diagnosis and treatment, and 3 questions on future research. For each question, statements with or without recommendations and/or evidence level are given and updated diagnostic and therapeutic algorithms are provided based on new evidence. Algorithms for diagnosis are requisite for patients with chronic abdominal pain or associated symptoms and/or abnormal bowel movement. Colonoscopy is indicated for patients with one or more alarm symptoms/signs, risk factors, and/or abnormal routine examination results. The diagnosis is based on the Rome IV criteria. Step 1 therapy consists of diet therapy, behavioral modification, and gut-targeted pharmacotherapy for 4 weeks. For non-responders, management proceeds to step 2 therapy, which includes a combination of different mechanistic gut-targeted agents and/or psychopharmacological agents and basic psychotherapy for 4 weeks. Step 3 therapy is for non-responders to step 2 and comprises a combination of gut-targeted pharmacotherapy, psychopharmacological treatments, and/or specific psychotherapy. These updated JSGE-IBS guidelines present best practice strategies for IBS patients in Japan and we believe these core strategies can be useful for IBS diagnosis and treatment globally.

## Introduction

Irritable bowel syndrome (IBS) is a prevalent disorder that greatly reduces patients’ quality of life (QOL) and adversely affects the medical economy [1]. A recent epidemiological survey using the Rome IV criteria revealed that the prevalence of IBS in the general population globally is 4.1% [2]. In Japan, the prevalence of IBS is 2.2% but that of functional bowel disorders is 25.2% [2]. Optimizing currently known treatments and developing new therapies for IBS are important efforts not only for patients themselves but also for society as a whole. The Japanese Society of Gastroenterology (JSGE) published the first edition of the clinical practice guidelines for IBS in Japanese in 2014 and in English in 2015 [3]. The guidelines were positively evaluated with the Appraisal of Guidelines for Research and Evaluation II (AGREE II) tool [4]. Following their publication, rapid advances in medical science and growing evidence of their utility led to the need to revise the JSGE-IBS guidelines 2014/2015. Here, we report the second version of the JSGE-IBS guidelines.

### Scope and purpose

The overall objectives of the guidelines are to provide evidence-based strategies to develop our understanding of the epidemiology, etiology, pathophysiology, and complications of IBS and for the diagnosis, treatment, and overall management of patients with IBS. Although the specific etiology of IBS is still unknown, treatment targeting a specific factor will help to estimate the contribution of that specific factor to IBS [5]. We designated the health questions as background questions (BQs), clinical questions (CQs), and future research questions (FRQs). BQs are questions with statements similar to those in the first edition of the guidelines regarding the epidemiology, etiology, pathophysiology, and complications, taking into account the accumulating evidence. CQs are questions with statements that either recommend or do not recommend diagnostic procedures or treatments with some level of evidence. FRQ are questions for which recommendations were not provided in the search for evidence. The target population for these guidelines is patients with IBS, over 15 years of age, visiting the clinic and/or hospital. However, some strategies may be applicable to non-patients with IBS or adolescent IBS patients. Specific subtypes of IBS (IBS-C, D, M, U) are described based on the Rome III [6] or IV [7] criteria.

### Stakeholder involvement

The guidelines development group included individuals from all relevant professional and geographically distributed groups mainly from the JSGE and additionally from the Japanese Gastroenterological Association, the Japanese Society of Neurogastroenterology and Motility, and the Japanese Society of Psychosomatic Medicine. These guidelines were developed after views and preferences were solicited from the target population via a web survey. The target users of these guidelines are gastroenterologists, internists, and general practitioners. These guidelines can also be used by psychosomaticists, geriatricians, psychiatrists, and psychologists.

### Rigor of development

Systematic methods were used to search for evidence [8]. In brief, we used the MEDLINE and Cochrane Library electronic databases to search for English articles published in the period from January 1983 to February 2019 and the *Igaku-chuou-zasshi* database for Japanese papers published in the period January 1983 to March 2019. Using specific keywords, 3031 references in English were identified for BQs, and 1918 references in English and 2220 references in Japanese were identified for the CQs and FRQs. Because some important articles were omitted partly because of the limits of the search, 16 papers were added to the review. Thus, 450 articles were included for analysis and 234 ones were listed in this article. The criteria for selecting the evidence were as follows: (A) systematic review, meta-analysis, or randomized controlled trial (RCT); (C) cohort study, case–control study; and (D) case series, case report, and expert opinion. We collected multiple articles from the literature and rated down or rated up the levels based on risk of bias or particular strengths. Thus, the level of evidence was assessed as high (A), moderate (B), low (C), or very low (D). Then the strength of recommendation was determined using the Grading of Recommendations Assessment, Development and Evaluation (GRADE) system [9, 10]. A statement with explanations about each CQ was made based on evidence with strong recommendation for or against or weak recommendation for or against the target procedure. The recommendations were formulated via a modified Delphi technique, with agreement needed from more than 70% of the development committee members upon voting. The health benefits, side effects, and risks were considered when formulating the recommendations as well as patient expectations and cost evaluation. There was an overt link between the recommendations and the supporting evidence. These guidelines were subjected to external review by experts prior to publication. These guidelines will be renewed in approximately 5 years.

### Clarity of presentation, applicability, and editorial independence

The recommendations are crafted to be specific and unambiguous, with different options for management of the condition or health issue clearly presented. Key recommendations of these guidelines are easily identifiable. These guidelines describe facilitators in their application (e.g., colonoscopy at high-volume colonoscopy centers) and barriers (cognitive behavioral therapy at colonoscopy centers). These guidelines also provide guidance and/or tools on how the recommendations can be put into practice. The potential resource implications of applying the recommendations (e.g., costs) were considered. These guidelines present monitoring and/or auditing criteria such as regular blood and fecal occult blood tests within 3 years after diagnosis. The funding body (JSGE) per se did not influence the content of these guidelines. Competing interests of the guideline development group members were recorded and addressed.

## Main text of the Japanese IBS guidelines

### Epidemiology and Pathophysiology

#### Epidemiology

BQ1-1. Is the prevalence of IBS increasing?

- **The prevalence of IBS is unlikely to be increasing.**
  Comment: A systematic review/meta-analysis in 2012 revealed the prevalence of IBS globally was 10% in 1981–1990, 12% in 1991–2000, and 11% in 2001–2010 [11]. The prevalence of IBS in Japan is not increasing in line with that worldwide [12, 13]. IBS is 1.6 times more frequent in females than in males [11] and prevalence decreases with age and differs among geographic regions (e.g., 2% in France and 21% in South America) [11].

BQ1-2. What do we understand about the prevalence and risk factors of post-infectious IBS (PI-IBS)?

- **PI-IBS develops in approximately 10% of patients with infectious enteritis due to known risk factors.**
  Comment: PI-IBS develops in approximately 10% of patients with infectious enteritis [14]. Risk factors identified include female sex, younger age, psychological distress during or before infectious gastroenteritis, and severity of enteritis [14]. Prevalence of IBS after infectious gastroenteritis or enterocolitis is reported to be 6–7 times higher than that without prior infectious episode [15]. The proportion of PI-IBS among all IBS cases is estimated to be 5%–25% [12, 16, 17]. Kanazawa et al. [12] reported that Japanese IBS patients and IBS nonconsulters were more likely to report an infective history compared with controls, supporting the notion that a history of acute gastroenteritis is a significant risk factor for the development of IBS in Japan, as reported in other countries.

#### Pathophysiology

BQ1-3. Is stress associated with the pathophysiology of IBS?

- **Stress is associated with the pathophysiology of IBS.**
  Comment: Aggravation of gastrointestinal symptoms was more strongly correlated with perceived stress in IBS patients than in healthy controls [18]. Colonic motility indices under stressful tasks were higher in IBS patients than in healthy controls [19]. Stress exacerbates activation of the right insula and left ventrolateral prefrontal cortex under rectal distension and inactivates the subgenual anterior cingulate cortex and right dorsolateral prefrontal cortex [20]. In a functional magnetic resonance imaging (fMRI) study, unpredictable visual stimulation before aversive rectal distension was activated more in the midcingulate cortex in IBS patients than in healthy controls [21]. Dynamic causal modeling analysis of fMRI in IBS patients revealed the inability to adjust appropriately to situational changes due to impaired activation in the right dorsolateral prefrontal cortex [22]. Also, negative events in adulthood were associated with symptom severity and altered stress response in IBS patients [23]. A meta-analysis showed that traumatic stress is a major risk factor for IBS with a pooled odds ratio of 2.8 [95% confidence interval of 2.06–3.54] [24].

BQ1-4. Are gut microbiota, increased mucosal permeability, and low-grade inflammation associated with the pathophysiology of IBS?

- **Gut microbiota, increased mucosal permeability, and low-grade inflammation are associated with the pathophysiology of IBS.**
  Comment: Gut microbiota, increased mucosal permeability, and low-grade inflammation are implicated in the pathophysiology of IBS [1]. These factors likely sensitize the neurons, which conduct signals from the gut to the central nervous system [1]. A systematic review clarified that approximately 10% of patients develop IBS after infectious gastroenteritis, with female sex, younger age, stress, and severity of acute episode as risk factors [14, 15]. Another systematic review of gut microbiota composition in IBS patients, based on 22 articles selected from among 2,631 studies [25], included a Japanese study that suggests some short-chain fatty acids have a role as gut microbiota metabolites [26]. The systematic review revealed decreased *Bifidobacterium* and *Faecalibacterium* and increased *Lactobacillaceae*, *Bacteroides*, and *Enterobacteriaceae* in IBS patients [25]. IBS patients have increased mucosal permeability regardless of a subtype with decreased expression of zonula occludens-1, α-catenin, and occludin as adhesion molecules of the gut epithelium as well as low-grade inflammation of the gut [27]. Cell populations in low-grade inflammatory infiltrate in the colonic mucosa of IBS patients consist of mast cells, eosinophils, macrophages, CD3 + cells, CD25 + cells, and intraepithelial lymphocytes [1, 15, 28]. Non-immunoglobulin E-related food allergy [28] and bile acid metabolism may also be involved in the various phenotypes of IBS [29, 30].

CQ1-5. Are neurotransmitters and endocrine substances involved in the pathophysiology of IBS?

- **Neurotransmitters and endocrine substances are involved in the pathophysiology of IBS.**
  Comment: A meta-analysis of brain imaging under colorectal distension in IBS patients showed hyperactivation of the anterior cingulate cortex, amygdala, and midbrain and deactivation of the medial and lateral prefrontal cortices [31]. In particular, IBS symptoms manifested as a centering association with functional network changes in the neurons of the amygdala [31]. Structural changes, specifically, decreased density, of the dorsolateral prefrontal cortex are noted in IBS patients [32], and the magnitude of decreased density is associated with loss of coping ability in response to stressors [33]. Some neurotransmitters and endocrine substances work in these regions of the brain as well as in the gut. Serotonin (5-hydroxytryptamine: 5-HT) plays major roles in diarrhea via 5-HT3 receptors [34] and in constipation via 5-HT4 receptors [35] in the gut and in anxiety via 5-HT3 receptors [36] and in abdominal pain via serotonin transporters [37] in the brain of IBS patients. Corticotropin-releasing hormone (CRH) is a key neuroendocrine factor involved in stress response, and the administration of CRH exaggerates colonic motility [38, 39] and plasma adrenocorticotropic hormone (ACTH) secretion [38–40] in IBS patients. The amygdala shows greater excitation following the administration of CRH in IBS patients than in healthy controls [41]. Plasma ACTH secretion induced by CRH administration is negatively correlated with pregenual anterior cingulate cortex activation in response to colorectal distension in healthy controls, but this response is disrupted in IBS patients [39]. Administration of the peptidergic CRH antagonist α-helical CRH suppresses stress-induced colonic motility, visceral pain, and anxiety in IBS patients [42]. Other substances including melatonin, histamine, glutamate via α2δ subunit of Ca^2+^ channels, and interleukin-6 (IL-6) have also been investigated [1, 3].

BQ1-6. Is psychological disturbance associated with the pathophysiology of IBS?

- **Psychological disturbance is associated with the pathophysiology of IBS.**
  Comment: Representative forms of psychological disturbance in IBS patients include depression, anxiety, and somatization [43]. Together with other psychological mechanisms like abuse, catastrophizing, and illness behaviors, these disturbances exacerbate the severity of IBS [43]. A 12-year cohort study showed that baseline depressive disorder or anxiety disorder was a risk factor for the new onset of IBS [44]. Inversely, functional gastrointestinal disorders including IBS and functional dyspepsia as a whole were reported to be risk factors for the new development of depressive disorder or anxiety disorder [44]. These phenomena imply pathophysiological brain-to-gut and gut-to-brain links.

BQ 1–7. Does genetics constitute the pathophysiology of IBS?

- **Genetics is involved in the pathophysiology of IBS.**
  Comment: The concordance rate of IBS in 6,060 twins was calculated [45] as 8.4% in dizygotic twins and 17.2% in monozygotic twins [45]. These data clearly indicate the hereditary nature of IBS. Despite the inclusion of acquired gender roles, a meta-analysis investigating sex differences clarified a female predominance of abdominal pain and constipation and male predominance of diarrhea [46], suggesting a possible chromosomal influence on IBS phenotype. Several candidate genes for IBS have been identified [1]. A cohort study of post-infectious IBS identified susceptible genes including IL-6, toll-like receptor 9 (TLR9), and E-cadherin-1 (CDH1) [47]. The G298S mutation of sodium channel Nav1.5 gene SCN5A was also detected in 13 of 584 patients with IBS (2.2%) [48]. Another meta-analysis of association studies of IBS with the tumor necrosis factor superfamily gene revealed an odds ratio 1.19 [95% confidence interval 1.08–1.31] [49]. A genome-wide association study in the EU and US identified KDEL endoplasmic reticulum protein retention receptor 2 (KDELR2) and glutamate receptor ionotropic delta 2 (Grid2) interacting protein (GRID2IP) genes in the short arm 22.1 of chromosome 7 [50]. Genetic studies on serotonin and CRH were also reported. Genotypes of serotonin transporter are given as *l/l, l/s,* and *s/s* graded in order from high to low activity of serotonin reuptake [51]. Subjects with the *s/s* gene show more exaggerated activity in the pregenual anterior cingulate cortex under colorectal distension than subjects with the *l/s* or *l/l* gene [51]. Brain network analysis also revealed that subjects with the *s/s* gene have more hippocampal input to the amygdala during colorectal distension than subjects with *l/s* or *l/l* genes [52]. Another meta-analysis showed a decreased risk of IBS in subjects with the *l/s* gene and increased risk of IBS-C in those with the *s/s* gene as well as populational differences between Asian and EU/US cohorts [53]. Some associations between IBS and CRH-related genes (CRH, CRH binding protein [54], CRH-R1 [55], and CRH-R2 [56]) were identified and replicated [57].

BQ 1–8. Does the pathophysiology of IBS differ among C, D, M, and U subtypes?

- **The pathophysiology of IBS differs among the C, D, M, and U subtypes, but a common pathophysiology is also seen.**
  Comment: There are phenotypic alterations in IBS [58]. Among female IBS patients followed up for 15 months, approximately 25% remained with the same subtype for over 12 months. The remaining 75% made a transition into at least one of the other subtypes [58]. A study measuring colonic transit time using radiopaque markers identified reasonably differing transit times among IBS-C, IBS-M, IBS-U, and IBS-D subtypes [59]. However, only 15% of IBS-C patients showed delayed transit time and only 36% of IBS-D patients showed rapid transit time [59]. Abdominal MRI revealed decreased small intestinal water content in IBS-D and IBS-M patients and increased volume in the transverse colon in IBS-C patients [60]. Principal component analysis of gut microbiota in IBS patients showed differences in the distribution patterns of IBS-C, IBS-M, and IBS-D [61]. Butyrate- and methane-producing bacteria were less abundant in IBS-D and IBS-M patients [61]. From an investigation of colonic motility and visceral perception measurements using manometry and barostat study, IBS patients showed more exaggerated colonic motility in response to colorectal distension and food intake and greater visceral hypersensitivity than healthy controls regardless of subtype [62]. These factors did not differ among subtypes [62]. These findings clearly depict the presence of both differential and common pathophysiology among subtypes in IBS.

## Diagnosis

BQ 2–1. Are the Rome IV criteria useful for diagnosis of IBS?

- **Rome IV criteria are useful for the diagnosis of IBS.**
  Comment: The Rome IV criteria [7] were derived from the Rome III criteria [6]. This revision was based on an accumulation of scientific evidence [2, 43]. Additional accumulation of evidence-based on Rome IV in the future will generate further scientific evidence for IBS treatment.
  CQ 2-1. Is colonoscopy necessary for the diagnosis of IBS?
- **Colonoscopy is useful for the differential diagnosis of IBS from other organic diseases. Histopathological examination of the gut mucosa is useful for differential diagnosis or identifying refractory IBS. We propose colonoscopy for the diagnosis of IBS. Weak recommendation, evidence level B, 100% agreed.**
  Comment: Colonoscopy offers diagnostic value to identify IBS patients and provide supportive evidence of pathophysiology compatible with IBS due to visceral hypersensitivity during colonoscopy as well as colonic dysmotility, with the additional benefit of excluding organic disease [63, 64]. Diagnostic colonoscopy in 4178 undiagnosed IBS patients showed that there were no differences in regard to the prevalence of organic colonic diseases between patients who did and did not fulfill the Rome III criteria, suggesting that these criteria cannot exclude organic colonic lesions [65]. Colonoscopy is necessary for IBS patients who have alarm signs/symptoms of organic disease. However, is should be noted that IBS patients may also have comorbid organic disease. Organic colonic lesions were found in 30.3% of patients with suspected IBS with no warning signs [66]. Histopathological examination of the gut mucosa is useful to exclude microscopic colitis, eosinophilic enteritis, and amyloidosis. Because microscopic colitis is diagnosed using histological criteria, a colonoscopy should be considered in cases of refractory IBS [67].

CQ 2–2. Are laboratory tests other than colonoscopy useful for differential diagnosis of IBS from organic diseases?

- **Upper gastrointestinal endoscopy, radiography, and specimen examination (blood, urine, and feces) are useful for differential diagnosis of IBS from organic diseases. Strong recommendation, evidence level B, 91% agreed.**
  Comment: Erythrocyte sedimentation rate (ESR) and C-reactive protein (CRP) measurements have been used to identify patients with inflammatory bowel disease (IBD). In studies using a value of 6 mg/L as a threshold CRP level, sensitivity was 77% and specificity was 70%. In studies using a value of 10 mm/h as threshold ESR level, sensitivity was 79% and specificity was 67% [68]. Calprotectin and lactoferrin tests have been used to identify patients with IBD. In a meta-analysis, CRP ≤ 0.5 and calprotectin ≤ 40 μg/g was found to essentially exclude IBD in patients with IBS symptoms while ESR and lactoferrin had little clinical utility [69]. Patients with celiac disease (CD) report symptoms similar to IBS. In another meta-analysis, the prevalence of biopsy-proven CD in cases that met the diagnostic criteria for IBS was more than fourfold that in controls without IBS [70]. The American Society for Gastrointestinal Endoscopy guidelines strongly recommends serum IgA tissue transglutaminase for CD screening [71]. Duodenal biopsy via upper gastrointestinal endoscopy is useful for confirmation of CD. However, the utility of screening tests for CD appears limited in east Asian countries including Japan because of the extremely low prevalence of CD [72]. A subset of patients with features compatible with IBS-D is those with bile acid malabsorption (BAM). In a meta-analysis study, 5 studies indicated that patients presenting with IBS-D symptoms include 10% with severe BAM, 17 studies indicated that patients presenting with IBS-D symptoms include 32% with moderate BAM, and 7 studies indicated patients presenting with IBS-D symptoms include 26% with moderate BAM [73]. The serum biomarkers 7α-hydroxy-4-cholesten-3-one and fibroblast growth factor-19 have been proposed as screening tests for BAM [74]. However, because these tests are not widely available, an alternative in practice is an empirical trial of bile acid sequestering agent therapy.

CQ 2–3. Are laboratory tests other than colonoscopy useful for identifying IBS?

- **Laboratory tests other than colonoscopy would not have sufficient diagnostic accuracy to identify IBS in routine use. However, because some tests can differentiate IBS from non-IBS with reasonable diagnostic accuracy, we propose these tests for the diagnosis of IBS. Weak recommendation, evidence level B, 100% agreed.**
  Comment: Ultrasonography has diagnostic value to identify IBS patients and evaluate specific intestinal or gallbladder motility patterns. In the postprandial phase, changes in the frequency of segmental contractions in the sigmoid colon were smaller in IBS-C patients; changes in the frequency of propulsion were larger in IBS-D patients [75]. IBS patients have increased gallbladder emptying compared with healthy subjects [76, 77]. Using fMRI, some patients with IBS can be detected based on visceral hypersensitivity seen as a painful response to rectal balloon-distension [78, 79]. Brain response to rectal balloon distension assessed by fMRI differed between patients with IBS-C and IBS-D [80]. Currently, no single serum biomarker can reliably differentiate IBS from organic disease. A case-control study investigated the predictive accuracy of a 10-biomarker algorithm for differentiating IBS from non-IBS; sensitivity and specificity were 50% and 88%, respectively [81]. Low sensitivity would render these tests inadequate for routine use. Fecal levels of chromogranins and secretogranins were associated with pathophysiological IBS phenotype [82]. More data will clarify the actual roles of these potential biomarkers of IBS.

CQ 2–4. Are laboratory tests useful during the clinical course of IBS?

- **Laboratory tests are useful during the clinical course of IBS. Strong recommendation, evidence level A, 100% agreed.**
  Comment: a systematic review of 14 studies on the natural history of IBS depicted 6 studies as reliable [83]. During the clinical course of IBS, the organic gastrointestinal disease was found in 2–5% of patients [83]. A cohort study with 57,851 IBS patients spanning 10 years revealed a standardized incidence ratio (SIR) of 8.42 [95% confidence interval 6.48–10.75] for colonic cancer and SIR 4.81 [95% confidence interval 2.85–7.60] for rectal cancer in the first 3 months [84]. However, the SIR of colorectal cancer over 4–10 years was consistently below 0.95 [84]. A retrospective 10-year observational study with 91,746 IBS patients and 182,492 controls revealed an increased risk of colorectal cancer in IBS during the initial 2 years, but this risk disappeared after 2 years [85]. If the diagnosis of IBS is accurate, IBS per se does not increase the risk of colorectal cancer [86]. IBS patients tend to have anxiety around developing cancer [86]. So, based on these data, laboratory tests at regular intervals are strongly recommended especially in the initial 3 years after the initial diagnosis of IBS.

## Treatment

CQ 3–1. Is dietary therapy effective in treating IBS?

- **Eliminating foods that exacerbate IBS symptoms, such as lipids, caffeine, spicy food, and milk and dairy products, is effective in managing IBS. Dietary therapy is recommended for IBS. Weak recommendation, evidence level B, 100% agreed.**
  Comment: Advice on regular dietary habits as a general measure may be required for most IBS patients. If symptoms worsen after taking a particular meal, eliminating culprit foods from the diet is necessary, such as foods with high-fat content, caffeine, spicy foods, and milk and dairy products. In western countries, several RCTs have revealed that a low fermentable, oligosaccharides, disaccharides, monosaccharides, and polyols (FODMAP) diet appears to be more effective than standard dietary advice for IBS patients [87, 88]. In Japan, evaluation of the low FODMAP diet has not shown clear advantage to date and requires further consideration.

CQ 3–2. Is behavioral modification other than change in diet effective in treating IBS?

- **Exercise therapy under proper instruction improves IBS symptoms. Weak recommendation, evidence level B, 92% agreed. There is no clear evidence for the utility of other behavioral modifications, such as eliminating alcohol and smoking or getting adequate sleep.**
  Comment: Twelve weeks of exercise significantly improved the symptoms and extraintestinal manifestations of IBS in 102 patients [89]. In the same intervention group, increased physical activity for an average observation period of 5.2 years had positive long-term effects on IBS symptoms [90]. In addition, a systematic review of 14 randomized studies reported that 1 h of yoga every day for 4 weeks, 0.5 h of walking almost every day for 12 weeks, and 0.5 to 1 h of aerobic exercise significantly improved IBS symptoms [91].

CQ3-3. Is bulking polymer or dietary fiber intake effective in treating IBS?

- **Bulking polymer intake or dietary fiber intake is an effective means of treating IBS. Bulking polymers or dietary fiber is recommended for IBS. Strong recommendation, evidence level A, 100% agreed.**
  Comment: Calcium polycarbophil is a hydrophilic polyacrylic resin but is insoluble in water. It functions under acidic conditions as soluble fiber by absorbing water and thus potentially improving stool consistency [92]. In a Japanese phase III randomized controlled study, polycarbophil calcium was superior to trimebutine maleate in efficacy and equal in safety [93]. Dietary fiber effectively improves the symptoms of IBS. An RCT comparing the efficacy of soluble fiber (psyllium, ispaghula), insoluble fiber (bran), and placebo in IBS patients revealed that soluble fiber significantly improved abdominal pain and discomfort compared with placebo [94]. A systematic review and meta-analysis confirmed the effect of soluble fiber in treating IBS [95].

CQ 3–4. Are gastrointestinal motility modifiers effective in treating IBS?

- **Gastrointestinal modifiers are effective in treating IBS. Gastrointestinal motility modifiers are recommended for IBS. Weak recommendation, evidence level B, 100% agreed.**
  Comment: Trimebutine maleate acts on the peripheral μ and κ opioid receptors [96] and is a representative gastrointestinal modifier [97]. The efficacy of trimebutine maleate in patients with IBS was investigated in several small-scale RCTs [98–101] and meta-analyses [102, 103] conducted overseas. This drug appears to improve gastrointestinal symptoms including abdominal pain in IBS patients, although no overall improvement was observed. The use of trimebutine maleate is generally recommended in some guidelines and reviews [104, 105]. With regard to dopamine D2 blocking agents, small-scale RCTs [106, 107] investigated the efficacy of domperidone in IBS patients and found no beneficial effect of this agent on gastrointestinal symptoms. No studies have investigated the utility of metoclopramide yet. Also, no clinical evidence is available on the efficacy of neostigmine or itopride in IBS patients.

CQ 3–5. Are anticholinergic agents effective in treating IBS?

- **Anticholinergic agents are effective in some patients with IBS. Anticholinergic agents are recommended for some patients with IBS. Weak recommendation, evidence level B, 100% agreed.**
  Comment: Anticholinergic agents have antispasmodic properties and are thought to be effective in the treatment of IBS. In Japan, tiquizium bromide, butylscopolamine bromide, timepidium bromide hydrate, and mepenzolate bromide have all been used as anticholinergic agents for the treatment of abdominal symptoms in IBS patients. In other countries, several small-scale RCTs [108–110] and meta-analyses [103, 111] of anticholinergic agents indicate that anticholinergic agents are effective in improving gastrointestinal symptoms including abdominal pain, although some reports do not appear to show improvement in overall symptoms [104, 112]. Anticholinergic agents available in Japan may be more appropriate for use in IBS treatment because of their slow-acting properties. In addition, side effects of anticholinergics such as thirst, constipation, and palpitation should be considered when using them [105].

CQ 3–6. Are probiotics effective in treating IBS?

- **Probiotics are effective in treating IBS. Probiotics are recommended for IBS. Strong recommendation, evidence level A, 100% agreed.**
  Comment: Probiotics are defined as live microorganisms that confer a significant health benefit to the host. The utility of probiotics in the treatment of IBS has been investigated in a large number of intervention studies including many high-quality systematic reviews, meta-analyses, and RCTs [113–122], but the results were somewhat inconsistent. Some studies with probiotics versus placebo found an improvement in global symptoms with probiotics, while others failed to demonstrate a clear effect of probiotics. This discrepancy in results may be attributable to methodological differences among trials, such as the type of probiotic used, duration of treatment, and outcome. Overall, probiotics are considered beneficial for IBS because of their relatively low cost and safety.

CQ 3–7. Are 5-HT3 receptor antagonists effective in treating IBS-D?

- **5-HT3 receptor antagonists are effective on IBS-D. 5-HT3 receptor antagonists are recommended for IBS-D. Strong recommendation, evidence level A, 100% agreed.**
  Comment: Systematic reviews and a network meta-analysis of RCTs confirmed that the 5-HT3 receptor antagonists (alosetron and ramosetron) significantly improved IBS-D symptoms, such as abdominal pain and discomfort in addition to defecation urgency, defecation frequency, and soft stool/diarrhea [123–125]. However, the use of alosetron is limited to specialist prescription and has been approved in the US only for female patients due to the complication of severe constipation and ischemic colitis [126–133]. An RCT showed ondansetron improved severity scores of IBS symptoms except pain scores compared with placebo [134]. In Japan, the efficacy of ramosetron 5 µg once daily was shown in multicenter double-blind RCTs, whereas treatment efficacy in female IBS-D patients was not fully proven [135, 136]. However, subsequent RCTs showed the benefit of ramosetron in female patients receiving half the above-mentioned dose of ramosetron [34, 137]. Currently approved doses are 5 µg-10 µg/day for men and 2.5 µg-5 µg/day for women; the other 5-HT3 receptor antagonists are not available for IBS-D in Japan.

CQ 3–8. Are anti-diarrheal agents effective on IBS-D?

- **Anti-diarrheal agents are effective in some patients with IBS-D. Anti-diarrheal agents are recommended for some patients with IBS-D. Weak recommendation, evidence level C, 100% agreed.**
  Comment: Antidiarrheal agents used in Japan include loperamide hydrochloride, albumin tannate, and berberine chloride. Several small-scale RCTs were conducted overseas to investigate the efficacy of loperamide in IBS-D patients [138–140], and the agent was found effective in improving defecation frequency and stool consistency. However, due to inconsistent results, no consensus has been reached on whether loperamide improves gastrointestinal symptoms such as abdominal pain. It should thus be used with caution due to the possibility of severe constipation and the addiction potential, and considering the US Food and Drug Administration (FDA) warning that it can cause serious heart problems. Although loperamide is often used as a first-line agent in patients with IBS-D, most guidelines suggest against continuous use [141–143]. Eluxadoline is a mixed μ and κ opioid receptor agonist and a *δ* opioid receptor antagonist; it has antidiarrheal and abdominal pain-modulating properties and notably does not cause profound constipation. Eluxadoline has been approved as a new therapeutic agent for IBS-D, after its benefit was confirmed in large-scale clinical trials [144, 145]. Furthermore, cholestyramine and colestimide, in addition to newer bile acid sequestrants such as colestipol and colesevelam, are bile acid sequestrants used for the treatment of BAM [146, 147]. A systematic review reported a high incidence of BAM among IBS-D patients [29]. These agents may have a role in the treatment of IBS-D, however, no RCT has been conducted in IBS-D patients and further evidence is required to confirm the role of BAM and the efficacy. Cholestyramine and colestimide are not officially approved for IBS-D, and eluxadoline and colesevelam are not available in Japan.

CQ 3–9. Are intestinal secretagogues effective for patients with IBS-C?

- **Intestinal secretagogues are effective and are recommended for use in patients with IBS-C. Strong recommendation, evidence level A, 100% agreed.**
  Comment: The intestinal secretagogues (gut epithelium modifiers) lubiprostone and linaclotide have been approved in Japan. Lubiprostone, a prostaglandin derivative, acts on the ClC-2 chloride channels of enterocytes. Linaclotide acts on enterocyte guanylate cyclase C (GC-C) receptors and activates the cystic fibrosis transmembrane conductance regulator via intra-cellular cGMP. These agents increase chloride secretion with sodium ions and water into the lumen, thereby accelerating intestinal transit. Additionally, linaclotide has an analgesic effect by inhibiting afferent visceral nerve activity through extra-cellular cGMP [148].
  In a meta-analysis, lubiprostone was effective for increasing spontaneous bowel movement (SBM), improving stool consistency and form and IBS-C symptoms such as abdominal pain, and fullness [149]. Safety and tolerability for long-term use are proven [150].
  Previous RCTs have shown a higher responder rate, that is, a decrease in abdominal pain and induction of complete SBM in the linaclotide group compared with the placebo group. A Japanese RCT also demonstrated higher effects of linaclotide (33.7%) compared with placebo (17.5%) on overall treatment improvement, with long-term efficacy [151]. A meta-analysis of GC-C receptor agonists demonstrated that linaclotide is significantly effective (OR: 2.43, CI: 1.43–3.98) compared with placebo [152]. A systematic review and network meta-analysis also showed the efficacy of four intestinal secretagogues (lubiprostone, linaclotide, plecanatide, and tenapanor) for IBS-C [153]. However, plecanatide and tenapanor are not available in Japan, and there are differences in the dosage of lubiprostone and linaclotide in various reports.

CQ 3–10. Are bile acids and an ileal bile acid transporter inhibitor effective for patients with IBS-C?

- **Bile acids and an ileal bile acid transporter inhibitor are suggested to be useful for patients with IBS-C. Weak recommendation, evidence level B, 92% agreed.**
  Comments: The primary bile acids (BAs) cholic acid (CA) and chenodeoxycholic acid (CDCA) are synthesized from cholesterol in hepatocytes. They are conjugated to glycine and taurine and secreted in the intestine. BAs act via transmembrane G protein-coupled receptor 5 (TGR5) present on the enterocytes and activate cystic fibrosis transmembrane, conductance regulator. Thus, BAs increase chloride secretion and water into the colon. BAs also act on TGR5 present on enterochromaffin cells, stimulate 5-HT release, and accelerate colonic peristalsis [154]. Total BAs, CDCA, and deoxycholic acid (DCA) content in feces showed positive correlations with colonic transit and stool frequency and form. Conversely, litocholic acid (LCA) showed a negative correlation with these parameters. IBS-C patients showed a significant decrease in total BA content in feces due to decreased BA synthesis and DCA, and increased proportions of fecal LCA compared with controls [155].
  In an RCT in female IBS-C patients, CDCA administration dose-dependently accelerated colonic transit and improved stool frequency and form compared with placebo [156]. Elobixibat interrupts the enterohepatic circulation of BAs, thereby upregulating hepatic BA synthesis. Thus, elobixibat can increase BA concentration in the colon and accelerate colonic transit. In an RCT in patients with chronic constipation, elobixibat improved stool frequency and form in IBS-C patients (30%) included in the study cohort [157]. Post-hoc analysis of these trials demonstrated a similar prevalence of abdominal pain between patients with and without IBS-C, and safety and long-term use tolerability [158]. However, elobixibat has not been approved for IBS-C patients.

CQ 3–11. Are 5-HT4 agonists effective in treating IBS-C?

- **5-HT4 agonists are effective in treating IBS-C. 5-HT4 agonists are recommended for IBS-C. Weak recommendation, evidence level B, 92% agreed.**
  Comment: At present, mosapride is the only 5-HT4 receptor agonist available for clinical use in Japan. This agonist is frequently used in Asian countries, especially in Japan, but rarely in the US and Europe. Mosapride improved rectosigmoid sensorimotor function [35]. Combination therapy with probiotics and mosapride was effective for the relief of symptoms [159]. The Japanese health insurance system covers the use of mosapride for chronic gastritis but not for IBS-C. In a meta-analysis, tegaserod 12 mg showed a higher relative risk (RR) of global relief in IBS-C patients than placebo and RR 1.54 (95% CI 1.35–1.75) indicated being a responder based on complete SBMs per week compared with placebo RR 0.6 (95% CI 0.42–0.78) in patients with chronic constipation [160]. It is indicated only for IBS-C in female patients under 65 years old in the US to avoid risk of ischemic heart disease. Prucalopride has been approved for clinical use in Europe based on evidence in patients with chronic constipation [161] but not in Japan.

CQ 3–12. Are non-stimulant (osmotic) laxatives effective in treating IBS-C?

- **Osmotic laxatives are effective for some patients with IBS-C. Osmotic laxatives are recommended for some patients with IBS-C. Weak recommendation, evidence level C, 100% agreed.**
  Comment: Magnesium oxide is used frequently in Japan. However, hypermagnesemia was reported when administered to patients with impaired renal function [162]; the Pharmaceuticals and Medical Devices Agency in Japan subsequently published pharmacological product safety information about magnesium oxide. The utility of polyethylene glycol in patients with chronic constipation and IBS-C has been shown in a meta-analysis and RCT [163–165]. The national health insurance system has covered the use of polyethylene glycol for chronic constipation since 2018. Lactulose and sorbitol are also used in the US and Europe. Only lactulose is indicated for chronic constipation in Japan.

CQ 3–13. Are stimulant laxatives effective in treating IBS-C?

- **Stimulant laxatives are effective in some patients with IBS-C. In principle, on-demand use of stimulant laxatives is recommended for some patients with IBS-C. Weak recommendation, evidence level D, 100% agreed.**
  Comment: No RCTs have investigated the effects of stimulant laxatives in patients with IBS only. Although stimulant laxatives clearly improve stool consistency and defecation frequency, their effects on abdominal pain and bloating as well as on QOL in IBS patients is currently unclear [166]. With regard to diphenylmethane laxatives like bisacodyl and sodium picosulfate, their utility in patients with chronic constipation has been shown in RCTs [167, 168]. However, caution should be exercised in the use, especially long-term use, of anthraquinone derivatives like senna, because of its negative aspects such as the development of tolerance, colon pigmentation or (pseudo-)melanosis coli, and abuse [166, 169, 170].

CQ 3–14. Are antidepressants useful for treating IBS?

- **Antidepressants are useful for IBS. Tricyclic antidepressants and selective serotonin reuptake inhibitors are recommended for patients with IBS depending on the pathophysiology, taking into consideration side effects. Weak recommendation, evidence level A, 92% agreed.**
  Comment: Antidepressants are used for IBS patients. IBS sometimes complicates depression and antidepressants have an effect on abdominal pain due to visceral hypersensitivity. There is much evidence of the effectiveness of tricyclic antidepressants (TCAs) and selective serotonin reuptake inhibitors (SSRIs) in treating IBS. In a meta-analysis of 15 placebo-controlled trials, TCAs and SSRIs significantly improved abdominal pain, general physical condition, and IBS severity score [104]. In the subgroup analysis, SSRIs improved general physical condition, while TCAs improved abdominal pain and IBS severity score. Although another meta-analysis of 12 RCTs found that TCAs significantly improved abdominal pain and general physical condition, SSRIs did not [171]. According to systematic reviews on the effect of antidepressants on IBS, although TCAs are effective especially in IBS-D patients, they often cause sleepiness, constipation, and dry mouth, causing many patients to withdraw from treatment [172]. Based on a meta-analysis of the side effects of antidepressants, SSRIs may be used safely for the most part in IBS-C [171]. On the other hand, no RCTs have been conducted to investigate the effects of serotonin/noradrenaline reuptake inhibitor (SNRI) use in IBS. In an open-label study [173], duloxetine was used in 15 IBS patients and significantly improved the severity of abdominal pain and symptoms overall, QOL, and anxiety. However, 7 of the 15 patients withdrew from the trial mostly due to constipation. In another RCT, the tetracyclic antidepressant mianserin significantly improved abdominal symptoms and social dysfunction related to functional gastrointestinal disorders (IBS and non-ulcer dyspepsia) compared with placebo [174]. Only one case report has mentioned noradrenergic and specific serotonergic antidepressants (NaSSA) for IBS therapy [175]. Although antidepressants, especially TCAs and SSRIs, are beneficial in treating IBS, they have various side effects. Antidepressants should thus be used in patients who fail to respond to standard therapy with due consideration of side effects. Physicians should consider the patient’s mental state when selecting antidepressants for drug therapy in IBS.

CQ 3–15. Are anxiolytics useful for treating IBS?

- **Anxiolytics are useful for treating IBS. Relieving anxiety is related to improving the symptoms of IBS in highly anxious patients. Anxiolytics are recommended for patients with IBS depending on the pathophysiology. Anxiolytics should be used for a short period while taking into account the risk of dependency. Weak recommendation, evidence level B, 100% agreed.**
  Comment: Anxiolytics, especially benzodiazepines, should be administered carefully because of risks of dependency. Because symptoms of IBS are often associated with anxiety, clinicians often prescribe anxiolytics for patients with IBS. However, investigations of the efficacy of a single anxiolytic are rare. Instead, several combination studies have been reported. In a double-blind study, the combined use of chlordiazepoxide and amitriptyline was more effective than antispasmodic, dietary fiber, or placebo [176]. These two drugs combined with an antispasmodic and dietary fiber were the most effective. In a multicenter double-blind study, the combined use of the antispasmodic octatropine and diazepam significantly improved abdominal pain and discomfort compared with placebo [177]. In an RCT of IBS-D, the combined use of the antispasmodic pinaverium and tandospirone significantly improved abdominal pain, discomfort, diarrhea, and anxiety after 8 weeks of administration compared with the antispasmodic pinaverium and placebo [178]. Tandospirone is a 5-HT_1A_ receptor agonist and anxiolytic, which is not categorized under benzodiazepines. Tandospirone is an effective alternative in treating IBS.

CQ 3–16. Is psychotherapy effective in treating IBS?

- **Psychotherapy is effective in treating patients with IBS. Psychotherapy is recommended for IBS patients. Strong recommendation, evidence level B, 100% agreed.**
  Comment: Psychotherapy includes cognitive-behavioral therapy (CBT) [179, 180], relaxation [181], hypnotherapy [182], mindfulness-based stress reduction (MBSR) [183], stress management [184], and psychodynamic therapy [185, 186]. A meta-analysis revealed that psychotherapy appears to be effective as treatment for IBS [187]. When all types of psychotherapeutic interventions were considered, the number needed to treat was 4 [187]. Standard CBT [179], relaxation therapy [181], hypnotherapy [182], psychodynamic therapy [185], and multi-component psychological therapy [188] were all more effective than control therapy [187]. Autogenic training is considered to be a type of self-induced hypnotherapy [189], and it has also been demonstrated to be more effective in the general improvement of IBS compared with control therapy (ie, supportive nutritional education) [189]. Because of a paucity of clinical trials, no beneficial effect has been detected for MBSR, stress management, or minimal contact CBT delivered via the internet in IBS yet [187]. Adverse events were poorly reported among trials of these various different psychotherapeutic interventions [187]. Another meta-analysis demonstrated that psychotherapy produced significantly greater improvements not only in gastrointestinal symptoms but also in mental health and functioning in daily activities in patients with IBS [190]. Of all types of psychotherapy, CBT produced the greatest improvements in daily functioning [190]. These findings have important implications for the treatment of IBS.
  Psychotherapy trials have methodological limitations because of the inability to blind patients or the investigators as to treatment assignment, and the difficulty of devising a placebo treatment that is credible but not effective. In addition, availability can be a problem in most primary care practices. Despite these limitations, psychotherapy is recommended for IBS patients who do not respond to standard pharmacological treatment.

CQ 3–17. Are *kampo* agents effective in treating IBS?

- ***Kampo* medicine (traditional Japanese medicine) is effective in treating IBS. Kampo agents are recommended for IBS. Weak recommendation, evidence level C, 100% agreed.**
  Comment: Traditional Japanese medicine, also called *kampo*, derives from traditional Chinese medicine. Few RCTs have been conducted on *kampo* agents (mainly herbal extract formations) for IBS [191]. Sasaki et al. reported that only abdominal pain was improved in a group of 232 IBS patients treated with herbal medicine containing *keishi-ka-shakuyaku-to* for 4 weeks [192]. It was noted that patients with diarrhea-predominant IBS assigned to the administration of *keishi-ka-shakuyaku-to* showed a significantly greater improvement of abdominal pain compared with those who received placebo. Adverse events were rarely reported throughout the study. However, the use of *kampo* agents is not highly recommended because of the overall low quality of the studies, the questionable manufacturing process of herbal medicines, and the lack of long-term follow-up. Because findings from some open-label and/or animal studies using several kinds of *kampo* agents have suggested benefits in improving IBS symptoms or pathophysiology [193–197], high-level RCTs are needed to further investigate their efficacy.

CQ 3–18. Are anti-allergic agents effective in treating IBS?

- **Anti-allergic agents are effective in treating IBS. Anti-allergic agents are recommended for in treating some patients with IBS. Strong recommendation, evidence level A, 83% agreed.**
  Comment: Food allergy has been proposed as one of the causes of IBS [198]. Eosinophilic gastroenteritis, which presents with abdominal pain like in IBS is also assumed to be associated with food allergy [199]. Therefore, accurate differential diagnosis is necessary. In a study of 409 IBS-D patients with positive skin prick tests, IBS symptoms improved significantly in both the elimination diet group and the anti-allergy medication (cromolyn) group [200]. In a double-blind RCT, compared with the placebo-treated group, IBS patients treated with the antiallergic drug ebastine for 12 weeks showed significant improvement of IBS symptoms [201]. However, no anti-allergic agents are currently approved for IBS treatment under the Japanese health insurance system.

CQ 3–19. Are antibiotics effective in treating IBS?

- **Some non-absorbable antimicrobial agents are effective as a treatment for IBS. Weak recommendation, evidence level A, 100% agreed.**
  Comment: In western countries, the efficacy of non-absorbable antimicrobial agents such as rifaximin or neomycin in the treatment of IBS has been proven by high-quality RCTs [202–206]. The proposed mechanism of this efficacy is the improvement of small intestinal bacterial overgrowth or some effect on gut microbiota. When the previous version of these guidelines were published [3], rifaximin was not approved in US or in Japan. Thus, we did not recommend antibiotics for use in IBS patients in the previous guidelines [3]. However, the FDA has since approved the administration of rifaximin in IBS patients. Moreover, under the Japanese health insurance system, rifaximin is approved for the treatment of hepatic encephalopathy with hyperammonemia. Therefore, we changed the statement with cautions as follows: (1) the use of rifaximin in IBS patients is not yet approved under the Japanese health insurance system; (2) the optimal dose of rifaximin for Japanese patients with IBS is unknown; and (3) pseudomembranous enteritis is a severe side effect of rifaximin.

CQ 3–20. Is comprehensive alternative medicine effective in treating IBS?

- **Peppermint oil is effective in treating IBS. Of all comprehensive alternative medicines, only peppermint oil is recommended for treating IBS. Weak recommendation, evidence level A, 100% agreed.**
  Comment: Peppermint oil is thought to alleviate IBS symptoms via calcium channel-mediated smooth muscle relaxation. Its efficacy in IBS has been shown in several RCTs, and in 4 meta-analyses, treatment outcome in patients administered peppermint oil was superior overall to outcome in the placebo group [207–210]. Many studies have investigated the effects of acupuncture in IBS. According to 2 meta-analyses, acupuncture improved the symptoms of IBS [211] and IBS-D [212] more than placebo, although caution is advised due to the study design.

CQ 3–21. Are narcotics and allied agents effective in alleviating abdominal pain in IBS?

- **Narcotics are not effective in alleviating abdominal pain in IBS. No narcotics are recommended for abdominal pain in IBS. Weak recommendation, evidence level C, 100% agreed.**
  Comment: Narcotics and allied agents (non-steroidal anti-inflammatory drugs, acetaminophen, and aspirin) are not effective in alleviating abdominal pain in IBS [213]. In fact, increasing the dosage of narcotics often causes chronic and recurrent abdominal pain (narcotic bowel syndrome) [214]. Although the effect is mediated via opioid receptors, eluxadoline is not classified as a narcotic (see CQ 3–8). In a pooled analysis of 3 RCTs that used the Rome III criteria to define IBS-D, eluxadoline was more effective than placebo (RR of IBS not improving 0.91; 95% confidence interval, 0.85–0.97) [209]. Eluxadoline was well tolerated in phase 2 [144] and 3 [145] trials, with constipation and nausea as the most common adverse events. The majority of serious adverse events (pancreatitis and sphincter of Oddi spasm) occurred in patients with pre-existing conditions including the absence of the gallbladder or advanced age [215].

CQ 3–22. Is it beneficial to prevent IBS patients from leaving without treatment?

- **IBS patients are at risk of many diseases and impaired QOL. It is beneficial to prevent IBS patients from leaving without treatment. Weak recommendation, evidence level C, 100% agreed.**
  Comment: IBS patients are at risk of self-injurious behavior [216] and developing IBDs [217], dementia [218], and Parkinson’s disease [219]. We therefore suggest performing or continuing medical intervention for IBS.

FRQ 3–1. Are anti-psychotics or mood stabilizers useful in treating IBS?

- **There is little evidence for the usefulness of anti-psychotics or mood stabilizers in patients with IBS. Anti-psychotics and mood stabilizers may be used in IBS patients to control abdominal pain or mental state in severe cases, but there are various side effects. Further studies are needed.**
  Comment: There is little evidence of the utility of anti-psychotics and mood stabilizers in treating IBS. In a case report, the atypical antipsychotic quetiapine was effective for severe refractory functional gastrointestinal disorders including IBS, but 10 of the 21 patients stopped using it because of side effects and ineffective relief of symptoms [220]. Anti-psychotics and mood stabilizers should be considered as an option for managing severe refractory cases [221]. These drugs should be administered carefully by well-experienced professionals.

FRQ 3–2. Is fecal microbiota transplantation (FMT) effective in IBS?

- **FMT is being investigated as a treatment for IBS. Further studies are needed to evaluate the efficacy of FMT in IBS.**
  Comments: In 2014, FMT for patients with IBS was reported for the first time globally [222]. This was reported for the first time in Japan in 2017 [223]. In that study, 6 IBS patients achieved clinical response, and the diversity of microbiota in patients with IBS was increased. In an RCT using feces from recipients themselves as a control group, the improvement rate was higher in the treatment group [224]. A randomized, double-blind, placebo-controlled trial of donor stool or placebo capsules in patients with IBS showed improved diversity of microbiota in the stool capsule group. However, the between-group improvement of symptoms was comparable [225]. In a meta-analysis of an RCT of FMT for IBS, symptom improvement in the 3 months after FMT was comparable with the placebo [226]. As mentioned above, FMT efficacy in IBS is as yet unclear. Although not limited to IBS, many factors such as route of administration, control group design, stool condition, donor stool origin, and the frequency of administration affect the results of FMT. Future large-scale studies or technological innovations regarding administration methods are expected.

FRQ 3–3. Are severity-dependent treatments more effective in treating IBS?

- **The concept of severity of IBS is clinically important, and treatment is also provided according to the severity of symptoms such as diarrhea, constipation, and abdominal pain. However, there are no reports on direct intervention studies comparing “treatment according to severity” with “treatment regardless of severity”. This is a focus for future study.**
  Comment: The concept of severity of IBS is clinically important and useful in making treatment planning decisions. Indicators such as the IBS-severity scoring system (IBS-SSS) are used in clinical trials [227], but no consensus has been reached. Treatment according to the severity of IBS symptoms such as diarrhea, constipation, and abdominal pain has been attempted [7]. However, there have been no reports of direct intervention studies comparing “treatment according to severity” with “treatment regardless of severity”. This is an issue for future study.

## Prognosis and complications

### Prognosis

BQ4-1. Does IBS affect survival rate, QOL, or healthcare-seeking behavior?

- **IBS affects QOL and healthcare seeking behavior.**
  Comment: Health-related QOL in patients with IBS is greatly impaired [228]. The severity of IBS (particularly abdominal pain or diarrhea) and psychological disturbance in IBS patients determine their healthcare-seeking behavior [229, 230]. Whether IBS actually impairs the survival rate is inconclusive, but the increased suicide rate in IBS patients should be recognized [216].

### Complications

BQ4-2. Does IBS show high co-morbidity with gastrointestinal diseases?

- **IBS patients show higher co-morbidity with functional dyspepsia, gastroesophageal reflux disease, or IBD than non-IBS patients.**
  Comment: In Japan, routine workplace health examinations revealed that the prevalence of functional dyspepsia or gastroesophageal reflux disease (GERD) in individuals with IBS was estimated at more than twofold that in individuals without IBS [231]. Symptoms compatible with IBS were significantly higher in patients with IBD compared with non-IBD controls [232]. The RR of transition from IBS to IBD was as high as 16.3 [217].

BQ4-3. Does IBS show high co-morbidity with extra-intestinal disorders?

- **IBS shows high co-morbidity with extra-intestinal disorders.**
  Comment: IBS shows high co-morbidity with extra-intestinal disorders such as fibromyalgia, chronic fatigue syndrome, chronic pelvic pain, temporomandibular joint disease, interstitial cystitis, premenstrual syndrome, bronchial asthma, dementia [218], Parkinson’s disease [219], and psychological disturbance especially anxiety and depression [233]. Almost all IBS patients who visit a clinic or hospital, or 18% of IBS subjects in the general population, have at least one or more psychological disturbance [233].

## Summary of questions, statements, and recommendation

Questions, statements, and recommendations on the IBS guidelines by the JSGE are summarized in Table 1. Major differences between the JSGE-IBS guidelines in 2014/2015 [3] and those in 2020 are as follows; (1) epidemiology, pathophysiology, prognosis, and complications were itemized as CQ in 2014/2015 but done as BQ in 2020. (2) Diagnostic criteria for IBS were renewed from Rome III to Rome IV with setting as BQ. (3) The recommendation of clinical examinations except for colonoscopy for differential diagnosis was weak in 2014/2015 but strong in 2020. (4) Recommendation of clinical examinations except for colonoscopy for IBS diagnosis was not present in 2014/2015 but weak with level B in 2020. (5) There was no description of laboratory tests during the clinical course in 2014/2015 but they were strongly recommended with evidence level A in 2020. (6) Evidence level of antidiarrheal agents for IBS-D was D in 2014/2015 but C in 2020. (7) Evidence level of intestinal secretagogues for IBS-C was B in 2014/2015 but A in 2020. (8) There was no description of bile acids or an ileal bile acid transporter inhibitor for IBS-C in 2014/2015 but they were weakly recommended with evidence level B in 2020. (9) The recommendation of psychotherapy was weak in 2014/2015 but strong in 2020. (10) Anti-allergics were strongly recommended with level B but weakly recommended with level A. (11) Antibiotics were not weakly recommended with level C in 2014/2015 but weakly recommended with level A in 2020. (12) Narcotics were not strongly recommended in 2014/2015 but not weakly done in 2020. (13) Antipsychotics/mood stabilizers, FMT, and severity dependent therapy were shown as FRQs in 2020 because there was no clear accumulation of conclusive evidence since 2014/2015. As more evidence was accumulated and new agents were developed in 5 years, the newer version of guidelines has higher quality than the older ones.

**Table 1:** Summarized questions and statements for irritable bowel syndrome JSGE clinical practice guidelines 2020

Q	Step	Subtype	Contents	Recommendation	Level
BQ1-1			Prevalence		
BQ1-2			Post-infectious IBS		
BQ1-3			Stress		
BQ1-4			Microbiota, mucosal permeability, low-grade inflammation		
BQ1-5			Neurotransmitters, endocrine substances		
BQ1-6			Psychological disturbances		
BQ1-7			Genetics		
BQ1-8			Pathophysiology among C, D, M, and U subtypes		
BQ2-1			Diagnosis based on Rome IV criteria		
CQ2-1			Colonoscopy	Weak	B
CQ2-2			Laboratory tests for differential diagnosis	Strong	B
CQ2-3			Some clinical tests for identifying IBS	Weak	B
CQ2-4			Laboratory tests for following-up	Strong	A
CQ3-1	1		Diet therapy	Weak	B
CQ3-2	1		Behavioral modification: Exercise	Weak	B
CQ3-3	1		Bulking polymer/dietary fiber	Strong	A
CQ3-4	1		Gastrointestinal motility modifiers	Weak	B
CQ3-5	1		Anticholinergics	Weak	B
CQ3-6	1		Probiotics	Strong	A
CQ3-7	1	D	5-HT3 receptor antagonists	Strong	A
CQ3-8	1	D	Anti-diarrheal agents	Weak	C
CQ3-9	1	C	Intestinal secretagogues	Strong	A
CQ3-10	1	C	Bile acids/ileal bile acid transporter inhibitor	Weak	B
CQ3-11	1	C	5-HT4 agonists	Weak	B
CQ3-12	1	C	Non-stimulant (osmotic) laxatives	Weak	C
CQ3-13	1	C	Stimulant laxatives, on-demand use	Weak	D
CQ3-14	2		Antidepressants	Weak	A
CQ3-15	2		Anxiolytics for a short period	Weak	B
CQ3-16	3		Psychotherapy	Strong	B
CQ3-17	1/2		Kampo	Weak	C
CQ3-18	1/2		Anti-allergics	Weak	A
CQ3-19	1/2		Antibiotics	Weak	A
CQ3-20	2		Comprehensive alternative medicine: peppermint oil	Weak	A
CQ3-21	2		Narcotics	Weak no	C
CQ3-22	1–3		Prevent patients from leaving without treatment	Weak	C
FRQ3-1	2/3		Anti-psychotics, mood stabilizer		
FRQ3-2	2/3		Fecal microbiota transplantation		
FRQ3-3	1–3		Severity-dependent treatment		
BQ4-1			Quality of life, medical seeking behavior		
BQ4-2			Co-morbidity with FD, GERD, IBD		
BQ4-3			Co-morbidity with extra-intestinal disorders		

*BQ* background question, *CQ* clinical question, *FRQ* future research question, *5-HT* 5-hydroxytryptamine, *FD* functional dyspepsia, *GERD* gastroesophageal reflux disease, *IBD* inflammatory bowel disease, *Level A* high evidence, *Level B* moderate evidence, *Level C* low evidence, *Level D* very low evidence, *Step 1* the first step of therapeutic algorithm, *Step 2* the second step of the therapeutic algorithm, *Step 3* the third step of the therapeutic algorithm, Subtype C: constipation-predominant IBS, *Subtype D* diarrhea-predominant IBS

## Diagnostic algorithm in IBS guidelines by the JSGE

**Fig. 1:**
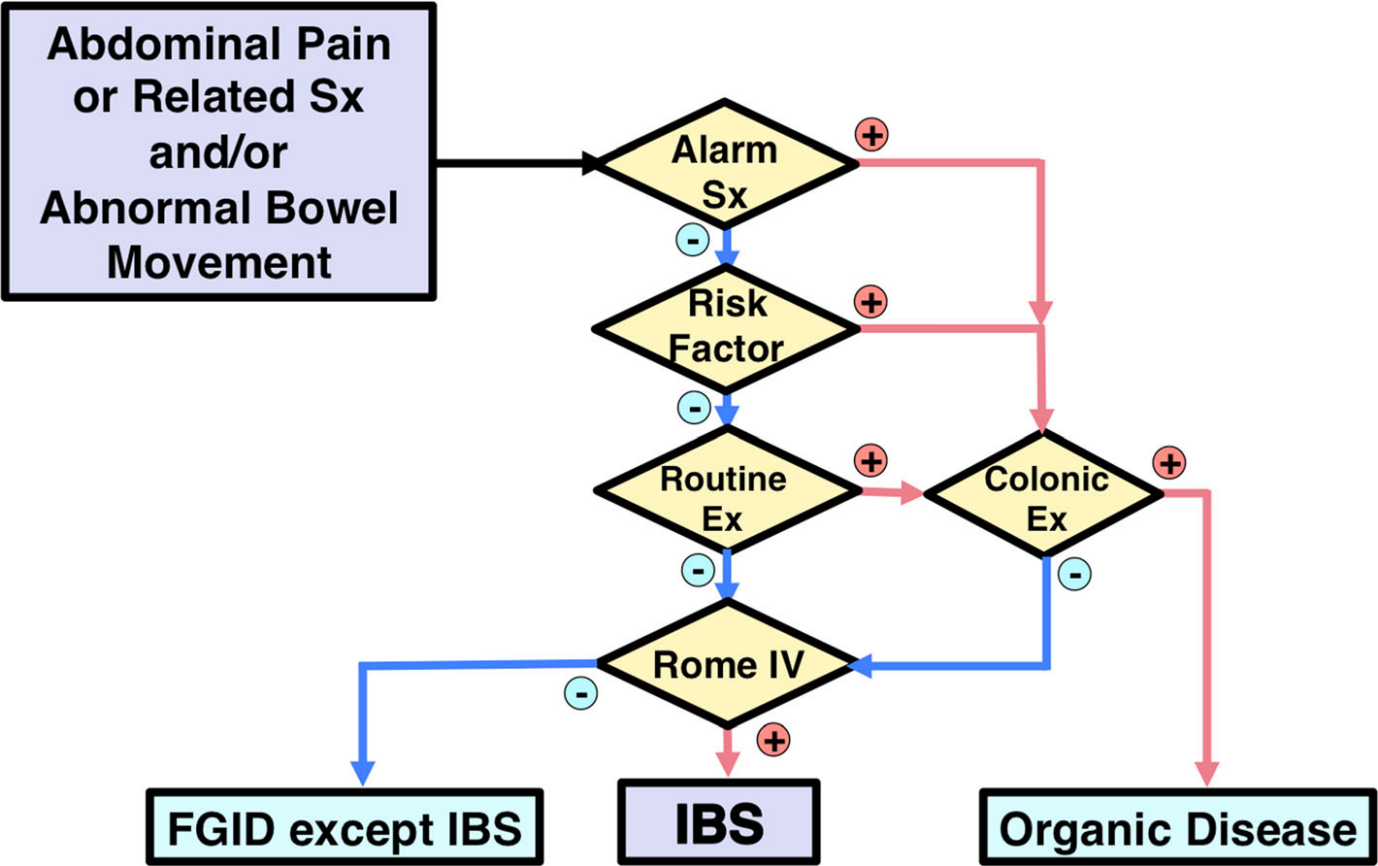
Diagnostic Algorithm for IBS. Check whether the answer is positive (yes) or negative (no) at the diamond. Alarm symptoms: bloody stool, unexpected weight loss more than 3 kg within 6 months, fever, and arthralgia. Alarm signs: palpable abdominal mass, abdominal fluctuation, palpable mass, or blood on the examining gloved finger on digital rectal examination. Risk factors: age over 50 years, past or family history of organic diseases of the colorectum, and patient’s requirement for colonic examination. Routine examinations: blood chemistry analyses including plasma glucose and thyroid-stimulating hormone, complete blood count, an inflammatory reaction such as (high-sensitive) C-reactive protein, urinalysis, fecal occult blood test, and plain abdominal X-ray. The colonic examination will be indicated if these factors are positive. Note that positive fecal occult blood, anemia, hypoproteinemia, or positive inflammatory reaction especially will require colonic examination. The colonic examination is mainly colonoscopy. It is the clinician’s responsibility to perform an adequate examination to reach an accurate diagnosis. The guidelines do not guarantee 100% exclusion of unexpected organic diseases. Depending on the clinical situation, the following examinations may be indicated: gastrointestinal mucosal biopsy, upper gastrointestinal endoscopy, barium enema, upper gastrointestinal series, abdominal ultrasonography, fecal ova test, stool bacterial culture, abdominal computed tomography, computed tomographic colonography, abdominal magnetic resonance imaging, small intestinal endoscopy, small intestinal fluoroscopy, lactose tolerance test, and hydrogen breath test. If clinical examinations results are negative and the Rome IV criteria are positive, a diagnosis of IBS is made. If the Rome IV criteria for IBS are negative, patients may be classified into other functional gastrointestinal disorders (FGIDs)

The JSGE guidelines propose a diagnostic algorithm for IBS (Fig. 1). Target patients are those with chronic (approximately more than 3 months) abdominal pain or related symptoms and/or abnormal bowel movement [3]. This diagnostic algorithm is not aimed to diagnose patients with acute GI symptoms. Colonoscopy is indicated if a patient has alarm symptoms/signs, risk factors, or abnormal routine examination results [63]. The Rome IV criteria are applied if colonoscopy and/or all of these are negative [7]. Diagnosis will be IBS or other functional gastrointestinal disorders like functional constipation, functional diarrhea, functional bloating, unspecified functional bowel disorder [7], or centrally mediated abdominal pain syndrome [214].

## Therapeutic algorithm in the IBS guidelines by the JSGE

**Fig. 2:**
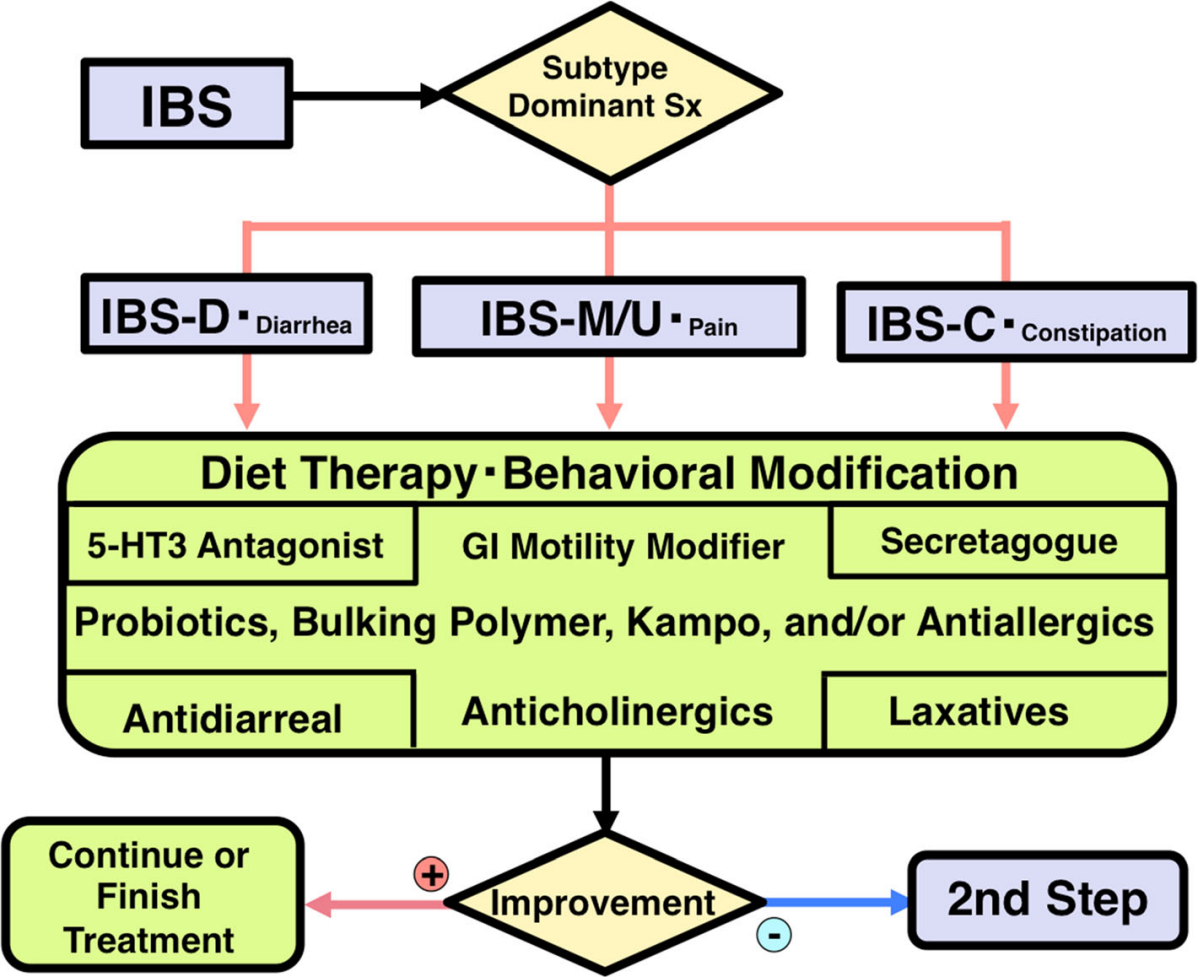
Step 1 of the IBS Therapeutic Algorithm. Subtyping of IBS is necessary at the time of treatment. Based on the Rome IV criteria, patients are classified as IBS with predominant diarrhea (IBS-D), IBS with mixed bowel habits (IBS-M), IBS unclassified (IBS-U), or IBS with predominant constipation (IBS-C). Moreover, the most bothersome symptoms including diarrhea, abdominal pain, or constipation may be targeted. See the main text for further details

Patients diagnosed with IBS are initially treated with step 1 therapy (Fig. 2). Dietary therapy such as the low FODMAP diet and behavioral modification including exercise [87–91] are indicated regardless of IBS subtype. Most patients need pharmacotherapy in addition to these modifications to lifestyle. Probiotics [113–122], bulking polymers [92, 93], and gastrointestinal motility modifiers [97–103] can be prescribed regardless of IBS subtype. For patients with IBS-D or with diarrhea as the main feature, 5-HT3 antagonists should be used [123–137]. For intractable cases, antidiarrheal agents including loperamide [138–140] or eluxadoline [144, 145] and bile acid sequestrants [146, 147] are the next in line for IBS-D. Intestinal secretagogues (gastrointestinal epithelium modifiers) [149–153, 234] are indicated for cases with IBS-C or constipation as the main feature. Laxatives [162–168] except for long-term use of anthraquinones [169, 170], bile acids [156], and ileal bile acid transporter inhibitors [157, 158] are the next in line for IBS-C. In IBS-M, IBS-U, or abdominal pain-dominant cases, anticholinergic agents can be used [103, 108–111]. In some cases, *kampo* [191–196], anti-allergic agents [200, 201], antibiotics [202–205], or peppermint oil [207–210] may be administered. After treatment with step 1 for 4 weeks, non-responders will advance to step 2.

**Fig. 3:**
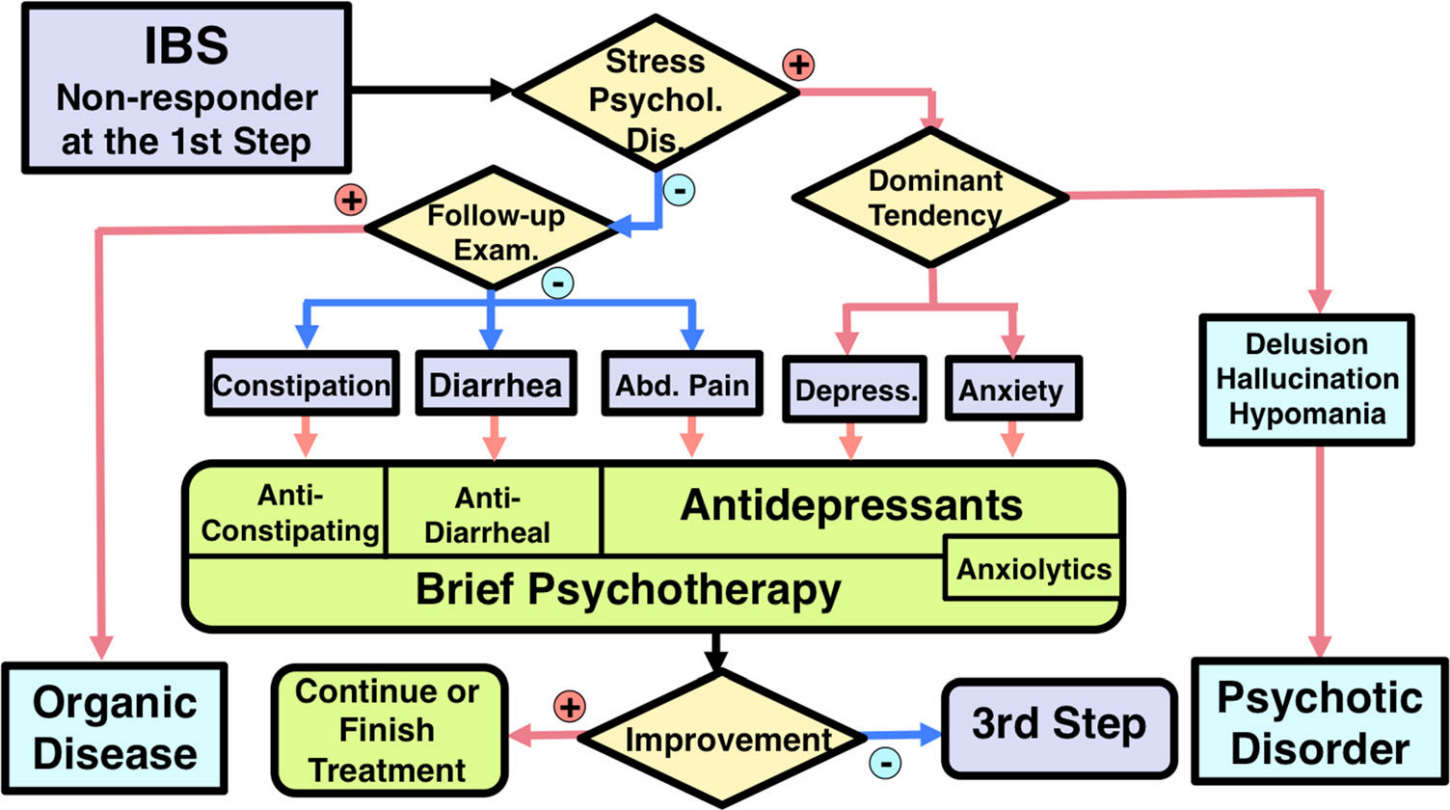
Step 2 of the IBS Therapeutic Algorithm. This step is indicated for IBS patients with moderate severity who do not respond to gut-targeted pharmacotherapy. For further details see the main text. Detailed examination described in the legend of Fig. 1 may be part of this step depending on the clinical demand

Step 2 therapy begins with an evaluation of the role of psychosocial stress [12–23] and co-morbid psychiatric diagnosis [24, 233] in each patient (Fig. 3). In patients with less influence from psychosocial factors, follow-up examinations and/or further gastrointestinal tract or other organ system examination [63–77] should be performed to rule out organic gastrointestinal or systemic disease. After confirmation of the accurate diagnosis of IBS, gastrointestinal agents that were not prescribed in step 1 therapy are indicated. Anti-constipation agents for constipation [149–153, 156–158, 162–168, 234], anti-diarrheal agents for diarrhea [123–147], and antidepressants for abdominal pain [171–175] are recommended. Antidepressants are indicated for IBS patients with depression [172, 187]. For IBS patients with anxiety, anxiolytic antidepressants are indicated [3, 187]. In these patients, anxiolytic drugs should mainly be prescribed from among 5-HT_1A_ agonists [178] or, if necessary, benzodiazepine derivatives and only for short durations [3, 177]. In some cases, brief psychotherapy [3, 180] can be added to manage psychosocial stress and negative emotion. If patients have delusion, hallucination, or hypomanic episodes, psychotic disorders should be suspected [3]. Thus, early collaboration with psychiatrists is indicated. The effect of step 2 therapy is evaluated for 4 weeks. Non-responders will proceed to step 3.

**Fig. 4:**
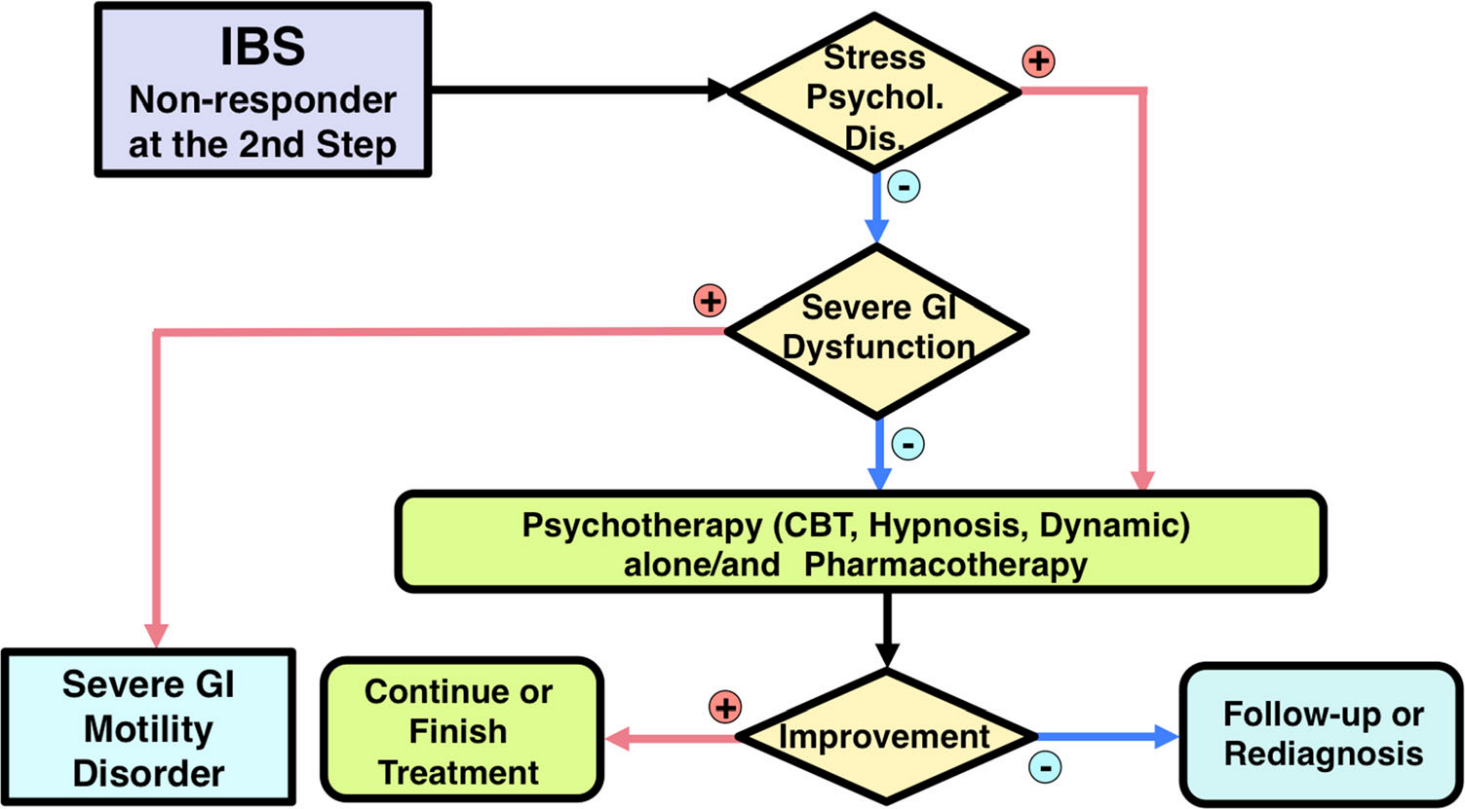
Step 3 of the IBS Therapeutic Algorithm. Severe IBS patients who do not respond to conventional pharmacotherapy are treated in this step. See the main text for further details. Gastrointestinal dysfunction can be determined with gastrointestinal transit study, anorectal manometry, colonic manometry, or colorectal barostat examination

Step 3 therapy begins with a repeat evaluation of the role of psychosocial stress or psychopathology in each patient. If negative, gastrointestinal imaging or motility examination [59–77] is indicated to determine coexisting pathophysiology of IBS such as mild lower gastrointestinal tract dysmotility or visceral hypersensitivity and/or to rule out severe gastrointestinal motility disorders. The majority of IBS patients usually have stress-related pathophysiology [18–24]. A combination of gastrointestinal agents, psychopharmacological treatments [171–175, 178, 187], and/or specific psychotherapy, particularly cognitive behavior therapy or hypnotherapy [179–190], will be helpful in these cases (Fig. 4). If patients do not respond to this level either, re-diagnosis or careful observation is required.

## Conclusion

The evidence-based clinical practice guidelines for IBS have been revised by the JSGE to reflect recent new evidence. The use of several strategies that are permitted in the management of IBS patients mainly in Japan have the potential to be applied globally. These clinical guidelines and consensus are the best applicable for IBS patients in Japan and we believe they can serve as a useful reference for IBS treatment worldwide.

## Appendix

The members of the Guidelines Committee who created and evaluated the Japanese Society of Gastroenterology ‘‘Evidence-based clinical guidelines for irritable bowel syndrome’’ are listed below.

## Creation Committee

Chair: Shin Fukudo (Department of Behavioral Medicine, Tohoku University Graduate School of Medicine).

Vice-Chair: Toshikatsu Okumura (Division of Gastroenterology and Hematology/Oncology, Department of Medicine, Asahikawa Medical University).

Members: Masahiko Inamori (Department of Medical Education, Yokohama City University School of Medicine), Yusuke Okuyama (Department of Gastroenterology, Japanese Red Cross Kyoto Daiichi Hospital), Motoyori Kanazawa (Department of Behavioral Medicine, Tohoku University GraduateSchool of Medicine), Takeshi Kamiya (Department of Gastroenterology and Metabolism, Nagoya City University), Ken Sato (Department of Gastroenterology and Hematology, Hirosaki University Graduate School of Medical Sciences), Akiko Shiotani (Department of Internal Medicine, Kawasaki Medical School), Yuji Naito (Department of Gastroenterology, Kyoto Prefectural University of Medicine), Yoshiko Fujikawa (Department of Gastroenterology, Higashisumiyoshi Morimoto Hospital), Ryota Hokari (Department of Gastroenterology, National Defense Medical College), Tastuhiro Masaoka (Department of Gastroenterology, Keio University School of Medicine)

## Evaluation Committee

Chair: Kazuma Fujimoto

(Department of Internal Medicine, International University of Health and Welfare, Graduate School of Medicine).

Vice-Chair: Hiroshi Kaneko (Department of Internal Medicine, Hoshigaoka Maternity Hospital).

Members: Akira Torii (Torii Clinic), Kei Matsueda (Sakura Life Kinshi Clinic)

## Japanese Society of Gastroenterology

President: Kazuhiko Koike (Department of Gastroenterology, Graduate School of Medicine, The University of Tokyo).

Past President: Tooru Shimosegawa (South Miyagi Medical Center).

Director Responsible: Hiroto Miwa (Division of Gastroenterology, Department of Internal Medicine, Hyogo College of Medicine), Nobuyuki Enomoto (First Department of Internal Medicine, Faculty of Medicine, University of Yamanashi).

## References

[1] Enck P, Aziz Q, Barbara G, Farmer A, Fukudo S, Mayer E, Niesler B, Quigley E, Rajilic-Stojanović M, Schemann M, Schwille-Kiuntke J, Simren M, Zipfel S, Spiller R (2016). Irritable bowel syndrome (IBS). Nat Rev Dis Primers.

[2] Sperber AD, Bangdiwala SI, Drossman DA, Ghoshal UC, Imren M, Tack J, Whitehead WE, Dumitrascu DL, Fang X, Fukudo S, Kellow J, Okeke E, Quigley EM, Schmulson M, Whorwell P, Archampong T, Adibi P, Andresen V, Benninga MA, Bonaz B, Bor S, Fernandez LB, Choi SC, Corazziari ES, Francisconi C, Hani A, Lazebnik L, Lee YY, Mulak A, Rahman MM, Santos J, Setshedi M, Syam AF, Vanner S, Wong RK, Lopez-Colombo A, Costa V, Dickman R, Kanazawa M, Keshteli AH, Khatun R, Maleki I, Poitras P, Pratap N, Stefanyuk O, Thomson S, Zeevenhooven J, Palsson OS (2020). Worldwide prevalence and burden of functional gastrointestinal disorders, results of Rome Foundation global study. Gastroenterology.

[3] Fukudo S, Kaneko H, Akiho H, Inamori M, Endo Y, Okumura T, Kanazawa M, Kamiya T, Sato K, Chiba T, Furuta K, Yamato S, Arakawa T, Fujiyama Y, Azuma T, Fujimoto K, Mine T, Miura S, Kinoshita Y, Sugano K, Shimosegawa T (2015). Evidence-based clinical practice guidelines for irritable bowel syndrome. J Gastroenterol.

[4] Li H, Xing X, Yao L, Li M, Xun Y, Yan P, Yang X, Yang K (2020). Assessment of the quality and content of clinical practice guidelines on irritable bowel syndrome using the AGREE II instrument. Digestion.

[5] Fukudo S (2013). Stress and visceral pain: focusing on irritable bowel syndrome. Pain.

[6] Longstreth GF, Thompson WG, Chey WD (2006). Functional bowel disorders. Gastroenterology.

[7] Lacy BE, Mearin F, Chang L, Chey WD, Lembo AJ, Simren M, Spiller R (2016). Bowel disorders. Gastroenterology.

[8] Yoshida M, Kinoshita Y, Watanabe M, Sugano K (2015). JSGE clinical practice guidelines 2014: standards, methods, and process of developing the guidelines. J Gastroenterol.

[9] Qaseem A, Snow V, Owens DK, Shekelle P (2010). Clinical Guidelines Committee of the American College of Physicians. The development of clinical practice guidelines and guidance statements of the American College of Physicians: summary of methods. Ann Intern Med.

[10] Qaseem A, Kansagara D, Lin JS, Mustafa RA, Wilt TJ (2019). Clinical Guidelines Committee of the American College of Physicians. The development of clinical guidelines and guidance statements by the clinical guidelines committee of the American College of Physicians: update of methods. Ann Intern Med.

[11] Lovell RM, Ford AC (2012). Global prevalence of and risk factors for irritable bowel syndrome: a meta-analysis. Clin Gastroenterol Hepatol.

[12] Kanazawa M, Endo Y, Whitehead WE (2004). Patients and nonconsulters with irritable bowel syndrome reporting a parental history of bowel problems have more impaired psychological distress. Dig Dis Sci.

[13] Kubo M, Fujiwara Y, Shiba M (2011). Differences between risk factors among irritable bowel syndrome subtypes in Japanese adults. Neurogastroenterol Motil.

[14] Barbara G, Grover M, Bercik P (2019). Rome Foundation working team report on post-infection irritable bowel syndrome. Gastroenterology.

[15] Thabane M, Kottachchi DT, Marshall JK (2007). Systematic review and meta-analysis: the incidence and prognosis of post-infectious irritable bowel syndrome. Aliment Pharmacol Ther.

[16] Porter CK, Gormley R, Tribble DR, Cash BD, Riddle MS, Porter CK (2011). The incidence and gastrointestinal infectious risk of functional gastrointestinal disorders in a healthy US adult population. Am J Gastroenterol.

[17] Longstreth GF, Hawkey CJ, Mayer EA (2001). Characteristics of patients with irritable bowel syndrome recruited from three sources: implications for clinical trials. Aliment Pharmacol Ther.

[18] Whitehead WE, Crowell MD, Robinson JC (1992). Effects of stressful life events on bowel symptoms: subjects with irritable bowel syndrome compared with subjects without bowel dysfunction. Gut.

[19] Fukudo S, Suzuki J (1987). Colonic motility, autonomic function, and gastrointestinal hormones under psychological stress on irritable bowel syndrome. Tohoku J Exp Med.

[20] Elsenbruch S, Rosenberger C, Bingel U (2010). Patients with irritable bowel syndrome have altered emotional modulation of neural responses to visceral stimuli. Gastroenterology.

[21] Kano M, Muratsubaki T, Morishita J (2017). Influence of uncertain anticipation on brain responses to aversive rectal distension in patients with irritable bowel syndrome. Psychosom Med.

[22] Aizawa E, Sato Y, Kochiyama T (2012). Altered cognitive function of prefrontal cortex during error feedback in patients with irritable bowel syndrome, based on fMRI and dynamic causal modeling. Gastroenterology.

[23] Parker CH, Naliboff BD, Shih W (2019). Negative events during adulthood are associated with symptom severity and altered stress response in patients with irritable bowel syndrome. Clin Gastroenterol Hepatol.

[24] Ng QX, Soh AYS, Loke W (2019). Systematic review with meta-analysis: the association between post-traumatic stress disorder and irritable bowel syndrome. J Gastroenterol Hepatol.

[25] Pittayanon R, Lau JT, Yuan Y (2019). Gut microbiota in patients with irritable bowel syndrome - A systematic review. Gastroenterology.

[26] Tana C, Umesaki Y, Imaoka A (2010). Altered profiles of intestinal microbiota and organic acids may be the origin of symptoms in irritable bowel syndrome. Neurogastroenterol Motil.

[27] Vivinus-Nébot M, Frin-Mathy G, Bzioueche H (2014). Functional bowel symptoms in quiescent inflammatory bowel diseases: role of epithelial barrier disruption and low-grade inflammation. Gut.

[28] Fritscher-Ravens A, Pflaum T, Mösinger M (2019). Many patients with irritable bowel syndrome have atypical food allergies not associated with immunoglobulin E. Gastroenterology.

[29] Slattery SA, Niaz O, Aziz Q (2015). Systematic review with meta-analysis: the prevalence of bile acid malabsorption in the irritable bowel syndrome with diarrhoea. Aliment Pharmacol Ther.

[30] Vijayvargiya P, Busciglio I, Burton D (2018). Bile acid deficiency in a subgroup of patients with irritable bowel syndrome with constipation based on biomarkers in serum and fecal samples. Clin Gastroenterol Hepatol.

[31] Tillisch K, Mayer EA, Labus JS (2011). Quantitative meta-analysis identifies brain regions activated during rectal distension in irritable bowel syndrome. Gastroenterology.

[32] Seminowicz DA, Labus JS, Bueller JA (2010). Regional gray matter density changes in brains of patients with irritable bowel syndrome. Gastroenterology.

[33] Blankstein U, Chen J, Diamant NE (2010). Altered brain structure in irritable bowel syndrome: potential contributions of pre-existing and disease-driven factors. Gastroenterology.

[34] Fukudo S, Kinoshita Y, Okumura T, Ida M, Akiho H, Nakashima Y, Nishida A, Haruma K (2016). Ramosetron reduces symptoms of irritable bowel syndrome with diarrhea and improves quality of life in women. Gastroenterology.

[35] Kanazawa M, Watanabe S, Tana C (2011). Effect of 5-HT4 receptor agonist mosapride citrate on rectosigmoid sensorimotor function in patients with irritable bowel syndrome. Neurogastroenterol Motil.

[36] Mayer EA, Berman S, Derbyshire SW (2002). The effect of the 5-HT3 receptor antagonist, alosetron, on brain responses to visceral stimulation in irritable bowel syndrome patients. Aliment Pharmacol Ther.

[37] Kułak-Bejda A, Bejda G, Waszkiewicz N (2017). Antidepressants for irritable bowel syndrome: a systematic review. Pharmacol Rep.

[38] Fukudo S, Nomura T, Hongo M (1998). Impact of corticotropin-releasing hormone on gastrointestinal motility and adrenocorticotropic hormone in normal controls and patients with irritable bowel syndrome. Gut.

[39] Kano M, Muratsubaki T, Van Oudenhove L (2017). Altered brain and gut responses to corticotropin-releasing hormone (CRH) in patients with irritable bowel syndrome. Sci Rep.

[40] Dinan TG, Quigley EM, Ahmed SM (2006). Hypothalamic-pituitary-gut axis dysregulation in irritable bowel syndrome: plasma cytokines as a potential biomarker?. Gastroenterology.

[41] Tanaka Y, Kanazawa M, Kano M (2016). Differential activation in amygdala and plasma noradrenaline during colorectal distention by administration of corticotropin-releasing hormone between healthy individuals and patients with irritable bowel syndrome. PLoS ONE.

[42] Sagami Y, Shimada Y, Tayama J (2004). Effect of a corticotropin releasing hormone receptor antagonist on colonic sensory and motor function in patients with irritable bowel syndrome. Gut.

[43] Drossman DA (2016). Functional gastrointestinal disorders: history, pathophysiology, clinical features and Rome IV. Gastroenterology.

[44] Koloski NA, Jones M, Kalantar J (2012). The brain-gut pathway in functional gastrointestinal disorders is bidirectional: a 12-year prospective population-based study. Gut.

[45] Levy RL, Jones KR, Whitehead WE (2001). Irritable bowel syndrome in twins: heredity and social learning both contribute to etiology. Gastroenterology.

[46] Adeyemo MA, Spiegel BM, Chang L (2010). Meta-analysis: do irritable bowel syndrome symptoms vary between men and women?. Aliment Pharmacol Ther.

[47] Villani AC, Lemire M, Thabane M (2010). Genetic risk factors for post-infectious irritable bowel syndrome following a waterborne outbreak of gastroenteritis. Gastroenterology.

[48] Saito YA, Strege PR, Tester DJ (2009). Sodium channel mutation in irritable bowel syndrome: evidence for an ion channelopathy. Am J Physiol Gastrointest Liver Physiol.

[49] Czogalla B, Schmitteckert S, Houghton LA (2015). A meta-analysis of immunogenetic case-control association studies in irritable bowel syndrome. Neurogastroenterol Motil.

[50] Ek WE, Reznichenko A, Ripke S (2015). Exploring the genetics of irritable bowel syndrome: a GWA study in the general population and replication in multinational case-control cohorts. Gut.

[51] Fukudo S, Kanazawa M, Mizuno T (2009). Impact of serotonin transporter gene polymorphism on brain activation by colorectal distention. Neuroimage.

[52] Kilpatrick LA, Mayer EA, Labus JS (2015). Serotonin transporter gene polymorphism modulates activity and connectivity within an emotional arousal network of healthy men during an aversive visceral stimulus. PLoS ONE.

[53] Zhu Y, Zheng G, Hu Z (2018). Association between SERT insertion/deletion polymorphism and the risk of irritable bowel syndrome: a meta-analysis based on 7039 subjects. Gene.

[54] Sasaki A, Sato N, Suzuki N (2016). Associations between single-nucleotide polymorphisms in corticotropin-releasing hormone-related genes and irritable bowel syndrome. PLoS ONE.

[55] Sato N, Suzuki N, Sasaki A (2012). Corticotropin-releasing hormone receptor 1 gene variants in irritable bowel syndrome. PLoS ONE.

[56] Komuro H, Sato N, Sasaki A (2016). Corticotropin-releasing hormone receptor 2 gene variants in irritable bowel syndrome. PLoS ONE.

[57] Orand A, Naliboff B, Gadd M (2016). Corticotropin-releasing hormone receptor 1 (CRH-R1) polymorphisms are associated with irritable bowel syndrome and acoustic startle response. Psychoneuroendocrinology.

[58] Drossman DA, Morris CB, Hu Y (2005). A prospective assessment of bowel habit in irritable bowel syndrome in women: defining an alternator. Gastroenterology.

[59] Törnblom H, Van Oudenhove L, Sadik R (2012). Colonic transit time and IBS symptoms: what’s the link?. Am J Gastroenterol.

[60] Lam C, Chaddock G, Marciani Laurea L (2017). Distinct abnormalities of small bowel and regional colonic volumes in subtypes of irritable bowel syndrome revealed by MRI. Am J Gastroenterol.

[61] Pozuelo M, Panda S, Santiago A (2015). Reduction of butyrate- and methane-producing microorganisms in patients with irritable bowel syndrome. Sci Rep.

[62] Kanazawa M, Palsson OS, Thiwan SI (2008). Contributions of pain sensitivity and colonic motility to IBS symptom severity and predominant bowel habits. Am J Gastroenterol.

[63] Kim ES, Cheon JH, Park JJ (2010). Colonoscopy as an adjunctive method for the diagnosis of irritable bowel syndrome: focus on pain perception. J Gastroenterol Hepatol.

[64] Mizukami T, Sugimoto S, Masaoka T (2017). Colonic dysmotility and morphological abnormality frequently detected in Japanese patients with irritable bowel syndrome. Intest Res.

[65] Ishihara S, Yashima K, Kushiyama Y (2012). Prevalence of organic colonic lesions in patients meeting Rome III criteria for diagnosis of IBS: a prospective multi-center study utilizing colonoscopy. J Gastroenterol.

[66] Gu HX, Zhang YL, Zhi FC (2011). Organic colonic lesions in 3,332 patients with suspected irritable bowel syndrome and lacking warning signs, a retrospective case: control study. Int J Colorectal Dis.

[67] Limsui D, Pardi DS, Camilleri M (2007). Symptomatic overlap between irritable bowel syndrome and microscopic colitis. Inflamm Bowel Dis.

[68] Dolwani S, Metzner M, Wassell JJ (2004). Diagnostic accuracy of faecal calprotectin estimation in prediction of abnormal small bowel radiology. Aliment Pharmacol Ther.

[69] Menees SB, Powell C, Kurlander J (2015). A meta-analysis of the utility of C-reactive protein, erythrocyte sedimentation rate, fecal calprotectin, and fecal lactoferrin to exclude inflammatory bowel disease in adults with IBS. Am J Gastroenterol.

[70] Ford AC, Chey WD, Talley NJ (2009). Yield of diagnostic tests for celiac disease in individuals with symptoms suggestive of irritable bowel syndrome: systematic review and meta-analysis. Arch Intern Med.

[71] Smalley W, Falck-Ytter C, Carrasco-Labra A (2019). AGA clinical practice guidelines on the laboratory evaluation of functional diarrhea and diarrhea-predominant irritable bowel syndrome in adults (IBS-D). Gastroenterology.

[72] Fukunaga M, Ishimura N, Fukuyama C (2018). Celiac disease in non-clinical populations of Japan. J Gastroenterol.

[73] Wedlake L, A’Hern R, Russell D (2009). Systematic review: the prevalence of idiopathic bile acid malabsorption as diagnosed by SeHCAT scanning in patients with diarrhoea-predominant irritable bowel syndrome. Aliment Pharmacol Ther.

[74] Valentin N, Camilleri M, Altayar O (2016). Biomarkers for bile acid diarrhoea in functional bowel disorder with diarrhoea: a systematic review and meta-analysis. Gut.

[75] Kusunoki H, Kamada T, Sato M (2006). Ultrasonographic assessment of sigmoid colon in patients with irritable bowel syndrome. Nihon Rinsho.

[76] Guliter S, Yilmaz S, Yakaryilmaz F (2005). Evaluation of gallbladder motility in patients with irritable bowel syndrome. Swiss Med Wkly.

[77] Güçlü M, Pourbagher A, Serin E (2006). Ultrasonographic evaluation of gallbladder functions in patients with irritable bowel syndrome. J Gastroenterol Hepatol.

[78] Yuan YZ, Tao RJ, Xu B (2003). Functional brain imaging in irritable bowel syndrome with rectal balloon-distention by using fMRI. World J Gastroenterol.

[79] Song GH, Venkatraman V, Ho KY, Chee MW, Yeoh KG, Wilder-Smith CH (2006). Cortical effects of anticipation and endogenous modulation of visceral pain assessed by functional brain MRI in irritable bowel syndrome patients and healthy controls. Pain.

[80] Guleria A, Karyampudi A, Singh R (2017). Mapping of brain activations to rectal balloon distension stimuli in male patients with irritable bowel syndrome using functional magnetic resonance imaging. J Neurogastroenterol Motil.

[81] Lembo AJ, Neri B, Tolley J (2009). Use of serum biomarkers in a diagnostic test for irritable bowel syndrome. Aliment Pharmacol Ther.

[82] Ohman L, Stridsberg M, Isaksson S (2012). Altered levels of fecal chromogranins and secretogranins in IBS: relevance for pathophysiology and symptoms?. Am J Gastroenterol.

[83] El-Serag HB, Pilgrim P, Schoenfeld P (2004). Systemic review: natural history of irritable bowel syndrome. Aliment Pharmacol Ther.

[84] Nørgaard M, Farkas DK, Pedersen L (2011). Irritable bowel syndrome and risk of colorectal cancer: a Danish nationwide cohort study. Br J Cancer.

[85] Hsiao CW, Huang WY, Ke TW (2014). Association between irritable bowel syndrome and colorectal cancer: a nationwide population-based study. Eur J Intern Med.

[86] Faresjo A, Grodzinsky E, Hallert C (2013). Patients with irritable bowel syndrome are more burdened by co-morbidity and worry about serious diseases than healthy controls: eight years follow-up of IBS patients in primary care. BMC Public Health.

[87] Staudacher HM, Whelan K, Irving PM (2011). Comparison of symptom response following advice for a diet low in fermentable carbohydrates (FODMAPs) versus standard dietary advice in patients with irritable bowel syndrome. J Hum Nutr Diet.

[88] Böhn L, Störsrud S, Liljebo T (2015). Diet low in FODMAPs reduces symptoms of irritable bowel syndrome as well as traditional dietary advice: a randomized controlled trial. Gastroenterology.

[89] Johannesson E, Simren M, Strid H (2011). Physical activity improves symptoms in irritable bowel syndrome: a randomized controlled trial. Am J Gastroenterol.

[90] Johannesson E, Ringström G, Abrahamsson H (2015). Intervention to increase physical activity in irritable bowel syndrome shows long-term positive effects. World J Gastroenterol.

[91] Zhou C, Zhao E, Li Y (2019). Exercise therapy of patients with irritable bowel syndrome: a systematic review of randomized controlled trials. Neurogastroenterol Motil.

[92] Toskes PP, Connery KL, Ritchey TW (1993). Calcium polycarbophil compared with placebo in irritable bowel syndrome. Aliment Pharmacol Ther.

[93] Masamune K, Miwa T, Fukutomi H (1998). Phase III trial of calcium polycarbophil in patients with irritable bowel syndrome: double-blind, randomized, controlled trial comparing trimebutine maleate. Yakuri-to-Chiryou.

[94] Bijkerk CJ, de Wit NJ, Muris JW (2009). Soluble or insoluble fibre in irritable bowel syndrome in primary care? Randomised placebo controlled trial. BMJ.

[95] Moayyedi P, Quigley EM, Lacy BE, Lembo AJ, Saito YA, Schiller LR, Soffer EE, Spiegel BM, Ford AC (2014). The effect of fiber supplementation on irritable bowel syndrome: a systematic review and meta-analysis. Am J Gastroenterol.

[96] Taniyama K, Sano I, Nakayama S (1991). Dual effect of trimebutine on contractility of the guinea pig ileum via the opioid receptors. Gastroenterology.

[97] Lee HT, Kim BJ (2011). Tromebutine as a modulator of gastrointestinal motility. Arch Pharm Res.

[98] Kang SH, Jeen YT, Koo JS (2013). Efficacy of fenoverine and trimebutine in the management of irritable bowel syndrome: multicenter randomized double-blind non-inferiority clinical study. Korean J Gastroenterol.

[99] Dumitrascu DL, Stanculete M (2006). The effect of trimebutine on the psychosocial adjustment to illness in the irritable bowel syndrome. Rom J Intern Med.

[100] Karabulut GS, Beser OF, Erginoz E (2013). The incidence of irritable bowel syndrome in children using the Rome III criteria and the effect of trimebutine treatment. J Neurogastroenterol Motil.

[101] Luttecke K (1980). A three-part controlled trial of trimebutine in the treatment of irritable colon syndrome. Curr Med Res Opin.

[102] Delvaux M, Wingate D (1997). Trimebutine: mechanism of action, effects on gastrointestinal function and clinical results. J Int Med Res.

[103] Poynard T, Naveau S, Mory B (1994). Meta-analysis of smooth muscle relaxants in the treatment of irritable bowel syndrome. Aliment Pharmacol Ther.

[104] Ruepert L, Quartero AO, de Wit NJ (2011). Bulking agents, antispasmodics and antidepressants for the treatment of irritable bowel syndrome cochrane database. Syst Rev.

[105] Heading R, Bardhan K, Hollerbach S (2006). Systematic review: the safety and tolerability of pharmacological agents for treatment of irritable bowel syndrome: a European perspective. Aliment Pharmacol Ther.

[106] Cann PA, Read NW, Holdsworth CD (1983). Oral domperidone: double blind comparison with placebo in irritable bowel syndrome. Gut.

[107] Fielding JF (1982). Domperidone treatment in the irritable bowel syndrome. Digestion.

[108] Khalif IL, Quigley EM, Makarchuk PA (2009). Interactions between symptoms and motor visceral sensory responses of irritable bowel syndrome patients to spasmolytics (antispasmodics). J Gastrointestin Liver Dis.

[109] Dobrilla G, Imbimbo BP, Piazzi L (1990). Long-term treatment of irritable bowel syndrome with cimetropium bromide: a double-blind placebo contolled clinical trial. Gut.

[110] Batttaglia G, Morselli-Labate AM, Camarri E (1998). Otilonium bromide in irritable bowel syndrome: a double-blinded, placebo-controlled, 15-week study. Aliment Pharmacol Ther.

[111] Ford AC, Talley NJ, Spiegel BM (2009). Effect of fibre, antispasmodics, and peppermint oil in the treatment of irritable bowel syndrome: a systematic review and meta-analysis. BMJ.

[112] Tack J, Fried M, Houghton LA (2006). Systematic review: the efficacy of treatments for irritable bowel syndrome- a European perspective. Aliment Pharmacol Ther.

[113] Brenner DM, Moeller MJ, Chey WD (2009). The utility of probiotics in the treatment of irritable bowel syndrome: a systematic review. Am J Gastroenterol.

[114] Moayyedi P, Ford AC, Talley NJ (2010). The efficacy of probiotics in the treatment of irritable bowel syndrome: a systematic review. Gut.

[115] Hoveyda N, Heneghan C, Mahtani KR (2009). A systematic review and meta-analysis: probiotics in the treatment of irritable bowel syndrome. BMC Gastroenterology.

[116] Spiller R (2008). Review article: probiotics and prebiotics in irritable bowel syndrome. Aliment Pharmacol Ther.

[117] Silk DBA, Davis A, Vulevic J (2009). Clinical trial: the effects of a trans-galactooligosaccharide prebiotic on faecal microbiota and symptoms in irritable bowel syndrome. Aliment Pharmacol Ther.

[118] Ford AC, Quigley EM, Lacy BE (2014). Efficacy of prebiotics, probiotics, and synbiotics in irritable bowel syndrome and chronic idiopathic constipation: systematic review and meta-analysis. Am J Gastroenterol.

[119] McKenzie YA, Thompson J, Gulia P (2016). British dietetic association systematic review of systematic reviews and evidence-based practice guidelines for the use of probiotics in the management of irritable bowel syndrome in adult (2016 update). J Hum Nur Diet.

[120] Hungin APS, Mitchell CR, Whorwell P (2018). Systematic review: probiotics in the management of lower gastrointestinal symptoms – an updated evidence-based international consensus. Aliment Pharmacol Ther.

[121] Barbara G, Cremon C, Azpiroz F (2018). Probiotics in irritable bowel syndrome: where are we?. Neurogastroenterol Motil.

[122] Ford AC, Harris LA, Jacy BE (2018). Systematic review with meta-analysis: the efficacy of prebiotics, probiotics, synbiotics and antibiotics in irritable bowel syndrome. Aliment Pharmacol Ther.

[123] Black CJ, Burr NE, Camilleri M (2019). Efficacy of pharmacological therapies in patients with IBS with diarrhoea or mixed stool pattern: systematic review and network meta-analysis. Gut.

[124] Zheng Y, Yu T, Tang Y (2017). Efficacy and safety of 5-hydroxytryptamine 3 receptor antagonists in irritable bowel syndrome: a systematic review and meta-analysis of randomized controlled trials. PLoS ONE.

[125] Andresen V, Montori VM, Keller J (2008). Effects of 5-hydroxytryptamine (serotonin) type 3 antagonists on symptom relief and constipation in nonconstipated irritable bowel syndrome: a systematic review and meta-analysis of randomized controlled trials. Clin Gastroenterol Hepatol.

[126] Cremonini F, Nicandro JP, Atkinson V (2012). Randomised clinical trial: alosetron improves quality of life and reduces restriction of daily activities in women with severe diarrhoea-predominant IBS. Aliment Pharmacol Ther.

[127] Chey WD, Chey WY, Heath AT (2004). Long-term safety and efficacy of alosetron in women with severe diarrhea-predominant irritable bowel syndrome. Am J Gastroenterol.

[128] Lembo T, Wright RA, Bagby B (2001). Lotronex investigator team. Alosetron controls bowel urgency and provides global symptom improvement in women with diarrhea-predominant irritable bowel syndrome. Am J Gastroenterol.

[129] Camilleri M, Chey WY, Mayer EA (2001). A randomized controlled clinical trial of the serotonin type 3 receptor antagonist alosetron in women with diarrhea-predominant irritable bowel syndrome. Arch Intern Med.

[130] Krause R, Ameen V, Gordon SH (2007). A randomized, double-blind, placebo-controlled study to assess efficacy and safety of 0.5 mg and 1 mg alosetron in women with severe diarrhea-predominant IBS. Am J Gastroenterol.

[131] Camilleri M, Mayer EA, Drossman DA (1999). Improvement in pain and bowel function in female irritable bowel patients with alosetron, a 5-HT3 receptor antagonist. Aliment Pharmacol Ther.

[132] Camilleri M, Northcutt AR, Kong S (2000). Efficacy and safety of alosetron in women with irritable bowel syndrome: a randomised, placebo-controlled trial. Lancet.

[133] Watson ME, Lacey L, Kong S (2001). Alosetron improves quality of life in women with diarrhea-predominant irritable bowel syndrome. Am J Gastroenterol.

[134] Garsed K, Chernova J, Hastings M (2014). A randomised trial of ondansetron for the treatment of irritable bowel syndrome with diarrhoea. Gut.

[135] Matsueda K, Harasawa S, Hongo M (2008). A randomized, double-blind, placebo-controlled clinical trial of the effectiveness of the novel serotonin type 3 receptor antagonist ramosetron in both male and female Japanese patients with diarrhea-predominant irritable bowel syndrome. Scand J Gastroenterol.

[136] Fukudo S, Ida M, Akiho H (2014). Effect of ramosetron on stool consistency in male patients with irritable bowel syndrome with diarrhea. Clin Gastroenterol Hepatol.

[137] Fukudo S, Kinoshita Y, Okumura T (2016). Effect of ramosetron in female patients with irritable bowel syndrome with diarrhea: a phase III long-term study. J Gastroenterol.

[138] Lavö B, Stenstam M, Nielsen AL (1987). Loperamide in treatment of irritable bowel syndrome: a double-blind placebo controlled study. Scand J Gastroenterol Suppl.

[139] Hovdenak N (1987). Loperamide treatment of the irritable bowel syndrome. Scand J Gastroenterol Suppl.

[140] Efskind PS, Bernklev T, Vatn MH (1996). A double-blind placebo-controlled trial with loperamide in irritable bowel syndrome. Scand J Gastroenterol.

[141] Faresjo A, Grodzinsky E, Johansson S (2008). Self-reported use of pharmaceuticals among patients with irritable bowel syndrome in primary care. J Manag Care Pharm.

[142] Hanauer SB (2008). The role of loperamide in gastrointestinal disorders. Rev Gastroenterol Disord.

[143] Videlock EJ, Chang L (2007). Irritable bowel syndrome: current approach to symptoms, evaluation, and treatment. Gastroenterol Clin North Am.

[144] Dove LS, Lembo A, Randall CW (2013). Eluxadoline benefits patients with irritable bowel syndrome with diarrhea in a phase 2 study. Gastroenterology.

[145] Lembo AJ, Lacy BE, Zuckerman MJ (2016). Eluxadoline for irritable bowel syndrome with diarrhea. N Engl J Med.

[146] Fernández-Bañares F, Rosinach M, Piqueras M (2015). Randomised clinical trial: colestyramine vs. hydroxypropyl cellulose in patients with functional chronic watery diarrhoea. Aliment Pharmacol Ther.

[147] Camilleri M, Acosta A, Busciglio I (2015). Effect of colesevelam on faecal bile acids and bowel functions in diarrhoea-predominant irritable bowel syndrome. Aliment Pharmacol Ther.

[148] Camilleri M, Ford AC (2017). Pharmacotherapy for irritable bowel syndrome. J Clin Med.

[149] Li F, Fu T, Tong WD (2016). Lubiprostone is effective in the treatment of chronic idiopathic constipation and irritable bowel syndrome: a systematic review and meta-analysis of randomized controlled trials. Mayo Clin Proc.

[150] Chey WD, Drossman DA, Johanson JF (2012). Safety and patient outcomes with lubiprostone for up to 52 weeks in patients with irritable bowel syndrome with constipation. Aliment Pharmacol Ther.

[151] Fukudo S, Miwa H, Nakajima A (2018). A randomized controlled and long-term linaclotide study of irritable bowel syndrome with constipation patients in Japan. Neurogastroenterol Motil.

[152] Shah ED, Kim HM, Schoenfeld P (2018). Efficacy and tolerability of guanylate cyclase-C agonists for irritable bowel syndrome with constipation and chronic idiopathic constipation: a systematic review and meta-analysis. Am J Gastroenterol.

[153] Black CJ, Burr NE, Quigley EMM (2018). Efficacy of secretagogues in patients with irritable bowel syndrome with constipation: systematic review and network meta-analysis. Gastroenterology.

[154] Bunnett NW (2014). Neuro-humoral signalling by bile acids and the TGR5 receptor in the gastrointestinal tract. J Physiol.

[155] Shin A, Camilleri M, Vijayvargiya P (2013). Bowel functions, fecal unconjugated primary and secondary bile acids, and colonic transit in patients with irritable bowel syndrome. Clin Gastroenterol Hepatol.

[156] Rao AS, Wong BS, Camilleri M (2010). Chenodeoxycholate in females with irritable bowel syndrome-constipation: a pharmacodynamic and pharmacogenetic analysis. Gastroenterology.

[157] Nakajima A, Seki M, Taniguchi S (2018). Safety and efficacy of elobixibat for chronic constipation: results from a randomised, double-blind, placebo-controlled, phase 3 trial and an open-label, single-arm, phase 3 trial. Lancet Gastroenterol Hepatol.

[158] Nakajima A, Taniguchi S, Kurosu S (2019). Efficacy, long-term safety, and impact on quality of life of elobixibat in more severe constipation: post hoc analyses of two phase 3 trials in Japan. Neurogastroenterol Motil.

[159] Choi CH, Kwon JG, Kim SK (2015). Efficacy of combination therapy with probiotics and mosapride in patients with IBS without diarrhea: a randomized, double-blind, placebo-controlled, multicenter, phase II trial. Neurogastroenterol Motil.

[160] Evans BW, Clark WK, Moore DJ (2007). Tegaserod for the treatment of irritable bowel syndrome and chronic constipation. Cochrane Database Syst Rev.

[161] Shin A, Camilleri M, Kolar G (2014). Systematic review with meta-analysis: highly selective 5-HT4 agonists (prucalopride, velusetrag or naronapride) in chronic constipation. Aliment Pharmacol Ther.

[162] Nyberg C, Hendel J, Nielsen OH (2010). The safety of osmotically acting cathartics in colonic cleansing. Nat Rev Gastroenterol Hepatol.

[163] Belsey JD, Geraint M, Dixon TA (2010). Systematic review and meta-analysis: polyethylene glycol in adults with non-organic constipation. Int J Clin Pract.

[164] Mínguez M, López Higueras A, Júdez J (2016). Use of polyethylene glycol in functional constipation and fecal impaction. Rev Esp Enferm Dig.

[165] Chapman RW, Stanghellini V, Geraint M (2013). Randomized clinical trial: macrogol/PEG 3350 plus electrolytes for treatment of patients with constipation associated with irritable bowel syndrome. Am J Gastroenterol.

[166] Khan S (2010). Diagnosis and management of IBS. Nat Rev Gastroenterol Hepatol.

[167] Kamm MA, Mueller-Lissner S, Wald A (2011). Oral bisacodyl is effective and well-tolerated in patients with chronic constipation. Clin Gastroenterol Hepatol.

[168] Mueller-Lissner S, Kamm MA, Wald A (2010). Multicenter, 4-week, double-blind, randomized, placebo-controlled trial of sodium picosulfate in patients with chronic constipation. Am J Gastroenterol.

[169] Wald A (2003). Is chronic use of stimulant laxatives harmful to the colon?. J Clin Gastroenterol.

[170] Alsalimy N, Madi L, Awaisu A (2018). Efficacy and safety of laxatives for chronic constipation in long-term care settings: a systematic review. J Clin Pharm Ther.

[171] Xie C, Tang Y, Wang Y (2015). Efficacy and safety of antidepressants for the treatment of irritable bowel syndrome: a meta-analysis. PLoS ONE.

[172] Friedrich M, Grady SE, Wall GC (2010). Effects of antidepressants in patients with irritable bowel syndrome and comorbid depression. Clin Ther.

[173] Brennan BP, Fogarty KV, Roberts JL (2008). Duloxetine in the treatment of irritable bowel syndrome: an open-label pilot study. Hum Psychopharmacol.

[174] Tanum L, Malt UF (1996). A new pharmacologic treatment of functional gastrointestinal disorder: a double-blind placebo-controlled study with mianserin. Scand J Gatroenterol.

[175] Akama F, Mikami K, Watanabe N, Kimoto K, Yamamoto K, Matsumoto H (2018). Efficacy of mirtazapine on irritable bowel syndrome with anxiety and depression: a case study. J Nippon Med Sch.

[176] Nigam P, Kapoor KK, Rastog CK (1984). Different therapeutic regimens in irritable bowel syndrome. J Assoc Physicians India.

[177] Pace F, Maurano A, Ciacci C (2010). Octatropine methyl bromide and diazepam combination (Valpinax) in patients with irritable bowel syndrome: a multicentre, randomized, placebo-controlled trial. Eur Rev Med Pharmacol Sci.

[178] Lan L, Chen YL, Zhang H (2014). Efficacy of tandospirone in patients with irritable bowel syndrome-diarrhea and anxiety. World J Gastroenterol.

[179] Lackner JM, Jaccard J, Krasner SS (2007). How does cognitive behavior therapy for irritable bowel syndrome work? A mediational analysis of a randomized clinical trial. Gastroenterology.

[180] Moss-Morris R, McAlpine L, Didsbury LP (2010). A randomized controlled trial of a cognitive behavioural therapy-based self-management intervention for irritable bowel syndrome in primary care. Psychol Med.

[181] Blanchard EB, Greene B, Scharff L, Schwarz-McMorris SP (1993). Relaxation training as a treatment for irritable bowel syndrome. Biofeedback Self Regul.

[182] Whorwell PJ, Prior A, Faragher EB (1984). Controlled trial of hypnotherapy in the treatment of severe refractory irritable-bowel syndrome. Lancet.

[183] Garland EL, Gaylord SA, Palsson O (2012). Therapeutic mechanisms of a mindfulness-based treatment for IBS: effects on visceral sensitivity, catastrophizing, and affective processing of pain sensations. J Behav Med.

[184] Shaw G, Srivastava ED, Sadlier M (1991). Stress management for irritable bowel syndrome: a controlled trial. Digestion.

[185] Svedlund J, Sjodin I, Ottosson JO, Dotevall G (1983). Controlled study of psychotherapy in irritable bowel syndrome. Lancet.

[186] Guthrie E (1991). Brief psychotherapy with patients with refractory irritable bowel syndrome. Br J Psychother.

[187] Ford AC, Lacy BE, Harris LA (2019). Effect of antidepressants and psychological therapies in irritable bowel syndrome: an updated systematic review and meta-analysis. Am J Gastroenterol.

[188] Neff DF, Blanchard EB (1987). A multi-component treatment for irritable bowel syndrome. Behav Ther.

[189] Shinozaki M, Kanazawa M, Kano M (2010). Effect of autogenic training on general improvement in patients with irritable bowel syndrome: a randomized controlled trial. Appl Psychophysiol Biofeedback.

[190] Laird KT, Tanner-Smith EE, Russell AC (2017). Comparative efficacy of psychological therapies for improving mental health and daily functioning in irritable bowel syndrome: a systematic review and meta-analysis. Clin Psychol Rev.

[191] Oka T, Okumi H, Nishida S, Ito T, Morikiyo S, Kimura Y, Murakami M (2014). JOPM-EBM working team effects of kampo on functional gastrointestinal disorders. Biopsycho Soc Med.

[192] Sasaki D, Uehara S, Hiwatashi N (1998). Clinical Efficacy of Keishi-ka-shakuyaku-to in patients with irritable bowel syndrome: a multicenter collaborative randomized controlled study Rinsho-to-Kenkyu. Japn J Clin Exp Med.

[193] Saitoh K, Kase Y, Ishige A, Komatsu Y, Sasaki H, Shibahara N (1999). Effects of Keishi-ka-shakuyaku-to (Gui-Zhi-Jia-Shao-Yao-Tang) on diarrhea and small intestinal movement. Biol Pharm Bull.

[194] Kase Y, Hayakawa T, Ishige A (1997). The effects of Hange-shashin-to on the content of prostaglandin E2 and water absorption in the large intestine of rats. Biol Pharm Bull.

[195] Bizen A (2012). Clinical efficacy of of hange-shashin-to in diarrhea-predominant irritable bowel syndrome patients with psychological stress: an open-label study Igaku-to- Yakugaku. Med Pharma.

[196] Manabe N, Camilleri M, Rao A (2010). Effect of daikenchuto (TU-100) on gastrointestinal and colonic transit in humans. Am J Physiol Gastrointest Liver Physiol.

[197] Nakaya K, Nagura Y, Hasegawa R (2016). Dai-Kenchu-To, a herbal Medicine, attenuates colorectal distention-induced visceromotor responses in rats. J Neurogastroenterol Motil.

[198] Nanda R, James R, Smith H (1989). Food intolerance and the irritable bowel syndrome. Gut.

[199] Kinoshita Y, Oouchi S, Fujisawa T (2019). Eosinophilic gastrointestinal diseases - Pathogenesis, diagnosis, and treatment. Allergol Int.

[200] Stefanini GF, Saggioro A, Alvisi V (1995). Oral cromolyn sodium in comparison with elimination diet in the irritable bowel syndrome, diarrheic type. Multicenter study of 428 patients. Scand J Gastroenterol.

[201] Wouters MM, Balemans D, Van Wanrooy S (2016). Histamine receptor H1-mediated sensitization of TRPV1 mediates visceral hypersensitivity and symptoms in patients with irritable bowel syndrome. Gastroenterology.

[202] Pimentel M, Park S, Mirocha J (2006). The effect of a nonabsorbed oral antibiotic (rifaximin) on the symptoms of the irritable bowel syndrome: a randomized trial. Ann Intern Med.

[203] Pimentel M (2010). An evidence-based treatment algorithm for IBS based on a bacterial/SIBO hypothesis: part 2. Am J Gastroenterol.

[204] Pimentel M, Lembo A, Chey WD (2011). Rifaximin therapy for patients with irritable bowel syndrome without constipation. N Engl J Med.

[205] Menees SB, Maneerattannaporn M, Kim HM, Chey WD (2012). The efficacy and safety of rifaximin for the irritable bowel syndrome: a systematic review and meta-analysis. Am J Gastroenterol.

[206] Pimentel M, Chatterjee S, Chow EJ (2006). Neomycin improves constipation-predominant irritable bowel syndrome in a fashion that is dependent on the presence of methane gas: subanalysis of a double-blind randomized controlled study. Dig Dis Sci.

[207] Cappello G, Spezzaferro M, Grossi L (2007). Peppermint oil (Mintoil) in the treatment of irritable bowel syndrome: a prospective double blind placebo-controlled randomized trial. Dig Liver Dis.

[208] Khanna R, MacDonald JK, Levesque BG (2014). Peppermint oil for the treatment of irritable bowel syndrome: a systematic review and meta-analysis. J Clin Gastroenterol.

[209] Moayyedi P, Andrews CN, MacQueen G (2019). Canadian Association of gastroenterology clinical practice guideline for the management of irritable bowel syndrome (IBS). J Can Assoc Gastroenterol.

[210] Alammar N, Wang L, Saberi B (2019). The impact of peppermint oil on the irritable bowel syndrome: a meta-analysis of the pooled clinical data. BMC Complement Altern Med.

[211] Wu IXY, Wong CHL, Ho RST (2019). Acupuncture and related therapies for treating irritable bowel syndrome: overview of systematic reviews and network meta-analysis. Therap Adv Gastroenterol.

[212] Zhu L, Ma Y, Ye S (2018). Acupuncture for diarrhoea-predominant irritable bowel syndrome: a network meta-analysis. Evid Based Complement Alternat Med.

[213] Chen L, Ilham SJ, Feng B (2017). Pharmacological approach for managing pain in irritable bowel syndrome: a review article. Anesth Pain Med.

[214] Keefer L, Drossman DA, Guthrie E, Simrén M, Tillisch K, Olden K, Whorwell PJ (2016). Centrally mediated disorders of gastrointestinal pain. Gastroenterology.

[215] Cash BD, Lacy BE, Schoenfeld PS (2017). Safety of eluxadoline in patients with irritable bowel syndrome with diarrhea. Am J Gastroenterol.

[216] Spiegel B, Schoenfeld P, Naliboff B (2007). Systematic review: the prevalence of suicidal behaviour in patients with chronic abdominal pain and irritable bowel syndrome. Aliment Pharmacol Ther.

[217] García Rodríguez LA, Ruigómez A, Wallander MA (2000). Detection of colorectal tumor and inflammatory bowel disease during follow-up of patients with initial diagnosis of irritable bowel syndrome. Scand J Gastroenterol.

[218] Chen CH, Lin CL, Kao CH (2016). Irritable bowel syndrome is associated with an increased risk of dementia: a nationwide population-based study. PLoS ONE.

[219] Lai SW, Liao KF, Lin CL, Sung FC (2014). Irritable bowel syndrome correlates with increased risk of Parkinson’s disease in Taiwan. Eur J Epidemiol.

[220] Grover M, Dorn SD, Weinland SR (2009). Atypical antipsychotic quetiapine in the management of severe refractory functional gastrointestinal disorders. Dig Dis Sci.

[221] Drossman DA (2009). Beyond tricyclics: new ideas for treating patients with painful and refractory functional gastrointestinal symptoms. Am J Gastroenterol.

[222] Pinn DM, Aroniadis OC, Brandt LJ (2014). Is fecal microbiota transplantation the answer for irritable bowel syndrome? A single-center experience. Am J Gastroenterol.

[223] Mizuno S, Masaoka T, Naganuma M (2017). Bifidobacterium-rich fecal donor may be a positive predictor for successful fecal microbiota transplantation in patients with irritable bowel syndrome. Digestion.

[224] Johnsen PH, Hilpüsch F, Cavanagh JP (2018). Faecal microbiota transplantation versus placebo for moderate-to-severe irritable bowel syndrome: a double-blind, randomised, placebo-controlled, parallel-group, single-centre trial. Lancet Gastroenterol Hepatol.

[225] Halkjaer SI, Christensen AH, Lo BZS (2018). Faecal microbiota transplantation alters gut microbiota in patients with irritable bowel syndrome: results from a randomised, double-blind placebo-controlled study. Gut.

[226] Xu D, Chen VL, Steiner CA (2019). Efficacy of fecal microbiota transplantation in irritable bowel syndrome: a systematic review and meta-analysis. Am J Gastroenterol.

[227] Drossman DA, Chang L, Bellamy N (2011). Severity in irritable bowel syndrome: a Rome foundation working team report. Am J Gastroenterol.

[228] Gralnek IM, Hays RD, Kilbourne A (2000). The impact of irritable bowel syndrome on health-related quality of life. Gastroenterology.

[229] Drossman DA, McKee DC, Sandler RS (1988). Psychosocial factors in the irritable bowel syndrome: a multivariate study of patients and nonpatients with irritable bowel syndrome. Gastroenterology.

[230] Talley NJ, Boyce PM, Jones M (1997). Predictors of health care seeking for irritable bowel syndrome: a population-based study. Gut.

[231] Kaji M, Fujiwara Y, Shiba M (2010). Prevalence of overlaps between GERD, FD and IBS and impact on health-related quality of life. J Gastroenterol Hepatol.

[232] Halpin SJ, Ford AC (2012). Prevalence of symptoms meeting criteria for irritable bowel syndrome in inflammatory bowel disease: systematic review and meta-analysis. Am J Gastroenterol.

[233] Whitehead WE, Palsson O, Jones KR (2002). Systematic review of the comorbidity of irritable bowel syndrome with other disorders: what are the causes and implications?. Gastroenterology.

[234] Fukudo S, Hongo M, Kaneko H (2011). Efficacy and safety of oral lubiprostone in constipated patients with or without irritable bowel syndrome: a randomized, placebo-controlled and dose-finding study. Neurogastroenterol Motil.

