# Correction: Evidence-based clinical practice guidelines for irritable bowel syndrome 2020

**DOI:** 10.1007/s00535-023-02044-0

**Published:** 2023-09-26

**Authors:** Shin Fukudo, Toshikatsu Okumura, Masahiko Inamori, Yusuke Okuyama, Motoyori Kanazawa, Takeshi Kamiya, Ken Sato, Akiko Shiotani, Yuji Naito, Yoshiko Fujikawa, Ryota Hokari, Tatsuhiro Masaoka, Kazuma Fujimoto, Hiroshi Kaneko, Akira Torii, Kei Matsueda, Hiroto Miwa, Nobuyuki Enomoto, Tooru Shimosegawa, Kazuhiko Koike

**Affiliations:** 1 Guidelines Committee for Creating and Evaluating the “Evidence-Based Clinical Practice Guidelines for Irritable Bowel Syndrome”, The Japanese Society of Gastroenterology, 6F Shimbashi I-MARK Building, 2-6-2 Shimbashi, Minato-Ku, Tokyo, 105-0004 Japan; 2 Department of Behavioral Medicine, Tohoku University Graduate School of Medicine, 2-1 Seiryo-Machi, Aoba-Ku, Sendai, 980-8575 Japan

**Correction: Journal of Gastroenterology (2020) 56:193–217** 10.1007/s00535-020-01746-z

The correct name of the co-author should be Tatsuhiro Masaoka, and not Tastuhiro Masaoka as given in the original publication of the article.

